# Spatial transcriptome-guided multi-scale framework connects *P*. *aeruginosa* metabolic states to oxidative stress biofilm microenvironment

**DOI:** 10.1371/journal.pcbi.1012031

**Published:** 2024-04-26

**Authors:** Tracy J. Kuper, Mohammad Mazharul Islam, Shayn M. Peirce-Cottler, Jason A. Papin, Roseanne M Ford

**Affiliations:** 1 Department of Chemical Engineering, University of Virginia, Charlottesville, Virginia, United States of America; 2 Department of Biomedical Engineering, University of Virginia, Charlottesville, Virginia, United States of America; University of Waterloo - Waterloo Campus: University of Waterloo, CANADA

## Abstract

With the generation of spatially resolved transcriptomics of microbial biofilms, computational tools can be used to integrate this data to elucidate the multi-scale mechanisms controlling heterogeneous biofilm metabolism. This work presents a Multi-scale model of Metabolism In Cellular Systems (MiMICS) which is a computational framework that couples a genome-scale metabolic network reconstruction (GENRE) with Hybrid Automata Library (HAL), an existing agent-based model and reaction-diffusion model platform. A key feature of MiMICS is the ability to incorporate multiple -omics-guided metabolic models, which can represent unique metabolic states that yield different metabolic parameter values passed to the extracellular models. We used MiMICS to simulate *Pseudomonas aeruginosa* regulation of denitrification and oxidative stress metabolism in hypoxic and nitric oxide (NO) biofilm microenvironments. Integration of *P*. *aeruginosa* PA14 biofilm spatial transcriptomic data into a *P*. *aeruginosa* PA14 GENRE generated four PA14 metabolic model states that were input into MiMICS. Characteristic of aerobic, denitrification, and oxidative stress metabolism, the four metabolic model states predicted different oxygen, nitrate, and NO exchange fluxes that were passed as inputs to update the agent’s local metabolite concentrations in the extracellular reaction-diffusion model. Individual bacterial agents chose a PA14 metabolic model state based on a combination of stochastic rules, and agents sensing local oxygen and NO. Transcriptome-guided MiMICS predictions suggested microscale denitrification and oxidative stress metabolic heterogeneity emerged due to local variability in the NO biofilm microenvironment. MiMICS accurately predicted the biofilm’s spatial relationships between denitrification, oxidative stress, and central carbon metabolism. As simulated cells responded to extracellular NO, MiMICS revealed dynamics of cell populations heterogeneously upregulating reactions in the denitrification pathway, which may function to maintain NO levels within non-toxic ranges. We demonstrated that MiMICS is a valuable computational tool to incorporate multiple -omics-guided metabolic models to mechanistically map heterogeneous microbial metabolic states to the biofilm microenvironment.

## Author summary

Microbes secrete and respond to environmental metabolite signals, resulting in the spatial organization of heterogeneous physiological metabolic states within infectious microbial communities. Despite experimental advances, it is difficult to measure the connected, dynamic processes that control microbial community organization across multiple spatial and time scales. Thus, we developed an extendable multi-scale computational framework to simulate metabolic processes spanning intracellular to extracellular scales. We used this framework to simulate a *Pseudomonas aeruginosa*, a bacterium that causes lung infections, 3D biofilm community containing tens of thousands of single-cells. This is the first framework to integrate spatially resolved gene expression data. This data integration was advantageous to simulate cells regulating anaerobic, toxic metabolite byproduct secretion, and antioxidant metabolic states in response to a heterogeneous oxygen and toxic byproduct biofilm microenvironment. These cellular metabolic states were predicted to co-exist in biofilm spatial niches due to microscale variations in extracellular oxygen and toxic metabolite signals. Difficult to measure in experiments, this framework revealed multi-scale mechanisms underlying the emergent dynamics of each metabolic state, which may help to inform *P*. *aeruginosa* biofilm treatment strategies. We believe this multi-scale framework is valuable to integrate microbial data to uncover the multi-scale mechanisms regulating heterogenous metabolic processes in microbial communities.

## Introduction

Biofilms are three-dimensional, dynamic, heterogeneous microbial communities. Emergent biofilm metabolite gradients result in the spatial organization of bacteria with distinct metabolic functions, impacting the fate of the microbial community to expand, cause infection, and resist antibiotics [1,2,3]. Gaining a mechanistic understanding of biofilm metabolic spatial organization is limited by current experimental tools, making it difficult to measure the connected intracellular and extracellular metabolic processes across multiple temporal and spatial scales [4]. For example, at single-cell resolution, spatial transcriptomic experiments retain the spatial locations of bacterial metabolic states within biofilms [5], but they do not couple that information with high-resolution metabolomics measurements that can reveal mechanistic relationships between metabolic states and metabolite microenvironments [6]. To overcome these current experimental limitations, computational tools that bridge mechanisms across temporal and spatial scales can reveal underlying intracellular and extracellular mechanisms and prioritize experiments to test potential therapeutic strategies to treat infectious biofilms [7,8].

One computational approach for simulating intracellular microbial metabolism uses genome-scale metabolic network reconstructions (GENREs). GENREs represent the complex, interconnected metabolic reaction network within a cell using a mathematical description of known gene-protein-reaction relationships and the stoichiometry of associated chemical transformations. GENREs can be interrogated with constraint-based flux-balance analysis (FBA) to simulate flux distributions associated with catabolic and anabolic processes [9]. Algorithms are being developed to integrate -omics data into a GENRE to constrain the intracellular metabolic solution space and generate biologically-relevant predictions of cellular metabolism in various metabolic environments [10–12].

Although -omics data integration algorithms enable improved prediction accuracy of cellular metabolism, outputs from FBA simulations often represent the steady-state metabolism of a given cell type or species and thus do not capture metabolic heterogeneity across space and time. Dynamic FBA can be used to predict temporal changes in biomass and extracellular metabolite concentrations, but lacks consideration of metabolic spatial heterogeneity [13]. To reveal metabolic differences in space, previous efforts integrated spatial transcriptomics data from healthy and cancerous tissue regions into a human GENRE, but lacked predictions of dynamic cell-cell and cell-environment interactions to predict disease dynamics [14]. Despite these efforts, few computational frameworks have simulated -omics-integrated GENREs in both spatial and temporal dimensions [15], which is important to mechanistically predict pathogenesis and therapeutic outcomes in addition to other biological process.

To simulate emergent spatiotemporal metabolic heterogeneity, computational frameworks have coupled GENREs with an agent-based model (ABM), which simulates individual cell behavior, and a reaction-diffusion model solved with partial differential equations (PDEs) that predicts extracellular metabolite concentrations. Previous 2D multi-scale frameworks, BacArena, MATNET, and COMETS, represent individual agents as a single cell or a population of cells, in which the simulation frameworks predicted emergent metabolic heterogeneity because each agent’s GENRE was constrained by local nutrient fluxes within a heterogenous nutrient environment [8,16,17]. Despite these efforts, few multi-scale frameworks are easily extendable for cells to dynamically adopt different -omics-integrated GENREs, which may improve predictions of metabolic processes controlled by gene regulation mechanisms. In addition, multi-scale frameworks have not incorporated GENREs integrated with spatial transcriptomic data, which can capture metabolic heterogeneity at single-cell spatial resolutions. For example, because the 3D multi-scale framework ACBM implemented a GENRE integrated with population-level transcriptomic data, this framework was not likely to capture potential biofilm metabolic heterogeneity measured at single-cell resolutions [15].

In this work, we present an extendable multi-scale computational framework that couples multiple -omics data-integrated GENREs, an ABM, and metabolite reaction-diffusion PDEs. We refer to this framework as a Multi-scale model of Metabolism In Cellular Systems (MiMICS). MiMICS is an open-source Java- and Python-based framework. MiMICS is extendable to simulate in 2D and 3D, and to represent individual agents as a single cell or a population of cells. A key feature of MiMICS is the ability to incorporate multiple -omics data-integrated GENREs, which can represent unique metabolic states. As a result of the corresponding integrated -omics data, each metabolic model state may predict different parameter values that alter the extracellular environment, such as nutrient uptake or toxic byproduct secretion. MiMICS allows for the user to incorporate multiple GENREs integrated with -omics data measured at the single-cell or population-scale level. While MiMICS was designed to integrate spatially resolved transcriptomics data, MiMICS could be used to integrate global transcriptomics data that was measured in various metabolic conditions. MiMICS can execute biologically-relevant ABM rules for cellular agents to choose from the -omics data-integrated GENREs to simulate metabolism. Simple mechanistic rules were used for a cell to switch metabolic model states related to the cell’s extracellular metabolic environment, effectively representing the transcription of metabolic genes regulated by the metabolic environment. Future studies could directly couple MiMICS with a gene regulatory network, which predicts gene transcription regulated by the metabolic environment [18].

As an initial biological test case to demonstrate its utility, MiMICS was applied to simulate emergent metabolic heterogeneity within a 3D *Pseudomonas aeruginosa* biofilm observed by a recent spatial transcriptomic study [5]. *P*. *aeruginosa* is an opportunistic pathogen that can cause deadly biofilm infections in the lungs of patients with cystic fibrosis and COVID-19 [19,20]. The published spatial transcriptomic study revealed microscale spatial organization of aerobic, denitrification, and oxidative stress metabolic states within a *P*. *aeruginosa* PA14 biofilm [5]. As proposed by Dar and co-workers [5], genes related to denitrification metabolism, an anaerobic respiration process, were hypothesized to be upregulated in anoxic PA14 biofilm regions [5, 21,22]. Likely secreted by denitrification cells, the cytotoxic denitrification intermediate nitric oxide (NO) was also hypothesized to upregulate oxidative stress genes in nearby PA14 biofilm cells [23]. However, the experiment lacked a quantitative and mechanistic mapping of the location of the cell and its metabolic state to the surrounding metabolic microenvironment[5]. Thus, herein, an established algorithm (RIPTiDe) [11] was used to integrate the published *P*. *aeruginosa* PA14 biofilm spatial transcriptomic dataset into a previously curated *P*. *aeruginosa* PA14 GENRE that generated four unique PA14 metabolic model states. The metabolic model states captured differences in aerobic and anaerobic denitrification metabolism, and revealed denitrification subpopulations that secreted the cytotoxic metabolite NO. This latter metabolic model state was crucial to predict a NO secretion rate that was passed to the extracellular reaction-diffusion model in MiMICS to generate a NO biofilm microenvironment that induced oxidative stress. Agents decided which metabolic model state to simulate their intracellular metabolic processes based on a combination of stochastic rules and metabolite sensing rules, the latter considering oxygen and NO levels in the agent’s local environment. MiMICS predicted microaerobic and variable NO microenvironments emerged within biofilm regions, resulting in microscale patches where cells heterogeneously used denitrification and oxidative stress metabolism. Due to cells sensing extracellular NO signals, MiMICS revealed the dynamics of cell populations heterogeneously regulating reactions in the denitrification pathway, which may function to maintain NO biofilm concentrations within non-toxic ranges. As demonstrated with this *P*. *aeruginosa* biofilm test case, we believe MiMICS is a promising computational tool that can use multiple -omics data-integrated metabolic models, and mechanistically simulate and map heterogeneous microbial metabolic states to the biofilm microenvironment.

## Results & discussion

**Fig 1:**
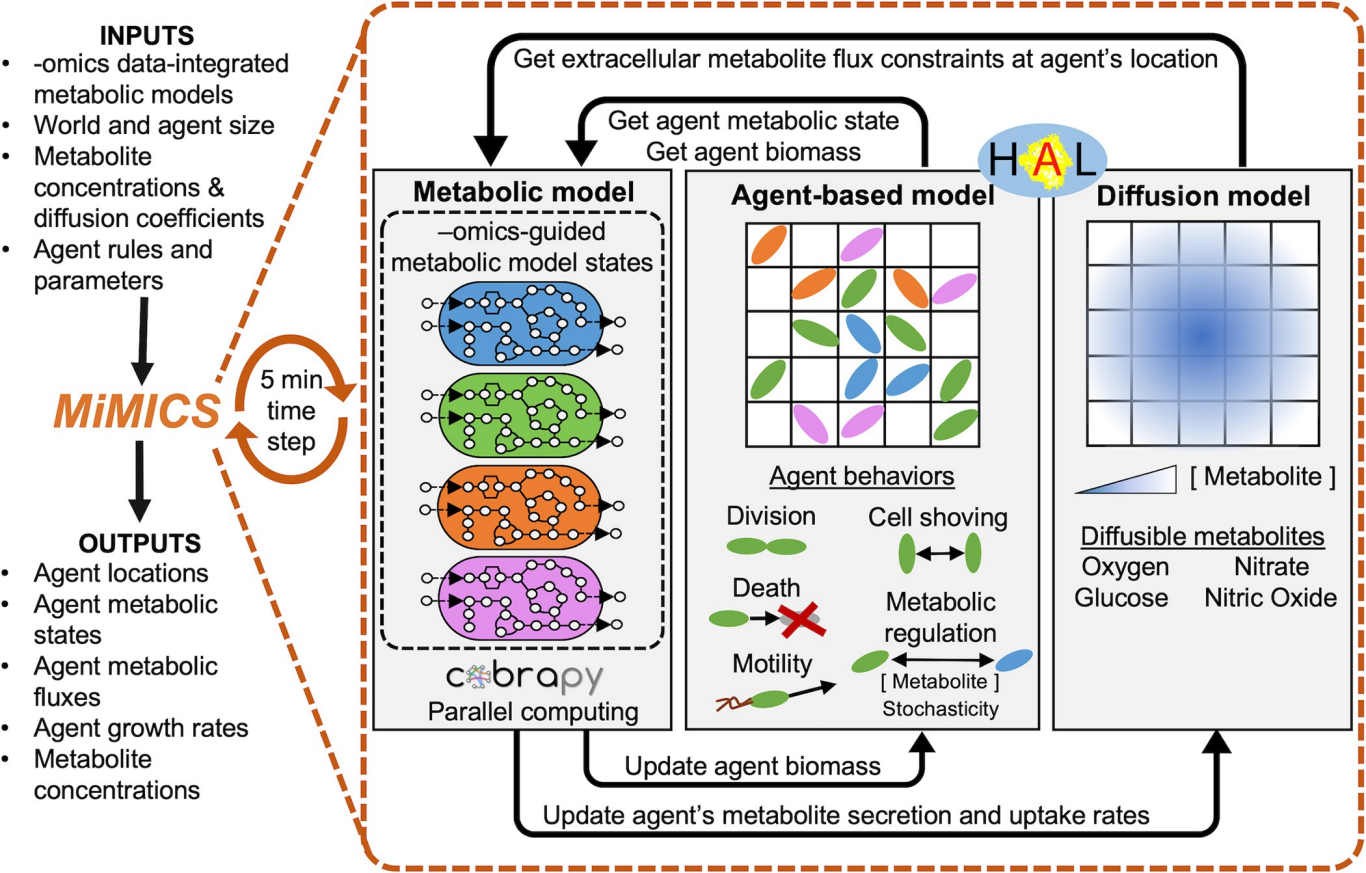
Overview of the MiMICS computational framework. Individual bacterial agents were initialized at t = 0hrs in a 3D ABM world. Metabolites were initialized based on user-defined metabolite concentrations. For each five-minute simulation time step, the MiMICS framework was executed, consisting of a set of–omics data-integrated metabolic models, an ABM, and a metabolite reaction-diffusion model. MiMICS simulation outputs included extracellular metabolite concentrations, as well as each agent’s location, metabolic state, and intracellular metabolite fluxes at each time point.

MiMICS is an extendable computational framework executed in Python and Java to simulate metabolism in 2D and 3D microbial communities. MiMICS couples a genome-scale metabolic network reconstruction (GENRE) with the established platform Hybrid Automata Library (HAL) [24], which contains an agent-based model (ABM) and a continuum-scale reaction-diffusion model (Fig 1). To our knowledge this is the first multi-scale metabolic framework to interface with the COBRApy Python package, which is becoming increasingly common to simulate and integrate -omics data into a GENRE [11,25,26]. In addition, MiMICS is the first framework to couple an intracellular metabolic model with HAL, and simulate 3D microbial biofilms using HAL, which can be challenging due to the small microbial length scales that can cause instability in PDE solvers [24]. MiMICS offers the user the ability to input multiple -omics data-integrated metabolic models, which can represent distinct metabolic states and yield different metabolite uptake or secretion rates that are passed to the extracellular reaction-diffusion model. Individual agents decide which metabolic model state to execute based on mechanistic rules input by the user, such as agents’ sensing of their local metabolite concentrations.

In this study, an individual 2 *μm* x 2 *μm* x 2 *μm* agent represented a single-cell *P*. *aeruginosa* bacterium, which existed on a 230 *μm* x 230 *μm* x 40 *μm* three-dimensional grid, corresponding to experimental microscopy dimensions [5]. The continuum-scale reaction-diffusion model simulated oxygen, nitrate, nitric oxide, and glucose concentrations within an equivalently sized three-dimensional metabolite grid solved with partial-differential equations [24]. Agents were randomly initialized at t = 0hrs, and MiMICS simulated biofilm growth for ten hours, replicating the experimental biofilm growth period [5]. At each five-minute simulation time step, each model component of MiMICS was executed to update agent properties and metabolite concentrations (Fig 1). For each agent, the agent’s biomass and metabolite concentrations from the continuum-scale grid corresponding to the agent’s location were converted to metabolite uptake fluxes used to constrain the agent’s intracellular metabolic model (Fig 1). Constraint-based flux-balance analysis was used to optimize each agent’s metabolic model to predict a biomass growth rate, as well as metabolite secretion and uptake rates. The biomass growth rate was passed to the ABM to update an agent’s biomass (Fig 1). Each agent’s predicted metabolite secretion and uptake fluxes were passed to the reaction-diffusion model to update the metabolite concentrations at each agent’s location (Fig 1). In the ABM, bacteria agents performed cell division, moved via motility, and performed cell mechanical behaviors to prevent cell overlap (Fig 1). Simulation outputs such as agent locations, agent intracellular metabolic fluxes and metabolite concentrations were reported at desired simulation time steps.

### Construction of spatial transcriptome-guided PA14 metabolic models

A key feature of MiMICS allows users to input multiple -omics-guided metabolic models, which may differ in predicted intracellular and extracellular metabolic parameter values, into a multi-scale metabolic framework (Fig 1). A set of four transcriptome-guided PA14 metabolic models was constructed by integrating an established PA14 GENRE [12] with a published spatial transcriptomic *P*. *aeruginosa* PA14 biofilm dataset [5] using the established algorithm RIPTiDe [11]. In addition, the PA14 GENRE was constrained on synthetic cystic fibrosis sputum medium (SCFM), replicating SCFM used in the experiment [5]. To avoid the computationally expensive generation of transcriptome-guided metabolic models for all ~292,000 PA14 biofilm cells measured, transcriptomic data representative of four unique metabolic states was extracted using the UMAP Leiden clustering method [5] and manual selection (*refer to Methods*). This approach generated four unique transcriptome-guided PA14 metabolic models. Each represented a unique metabolic state within the ten-hour PA14 biofilm, designated as (1) aerobic respiration, (2) denitrification, (3) denitrification + nitric oxide (NO) secretion, and (4) oxidative stress.

**Fig 2:**
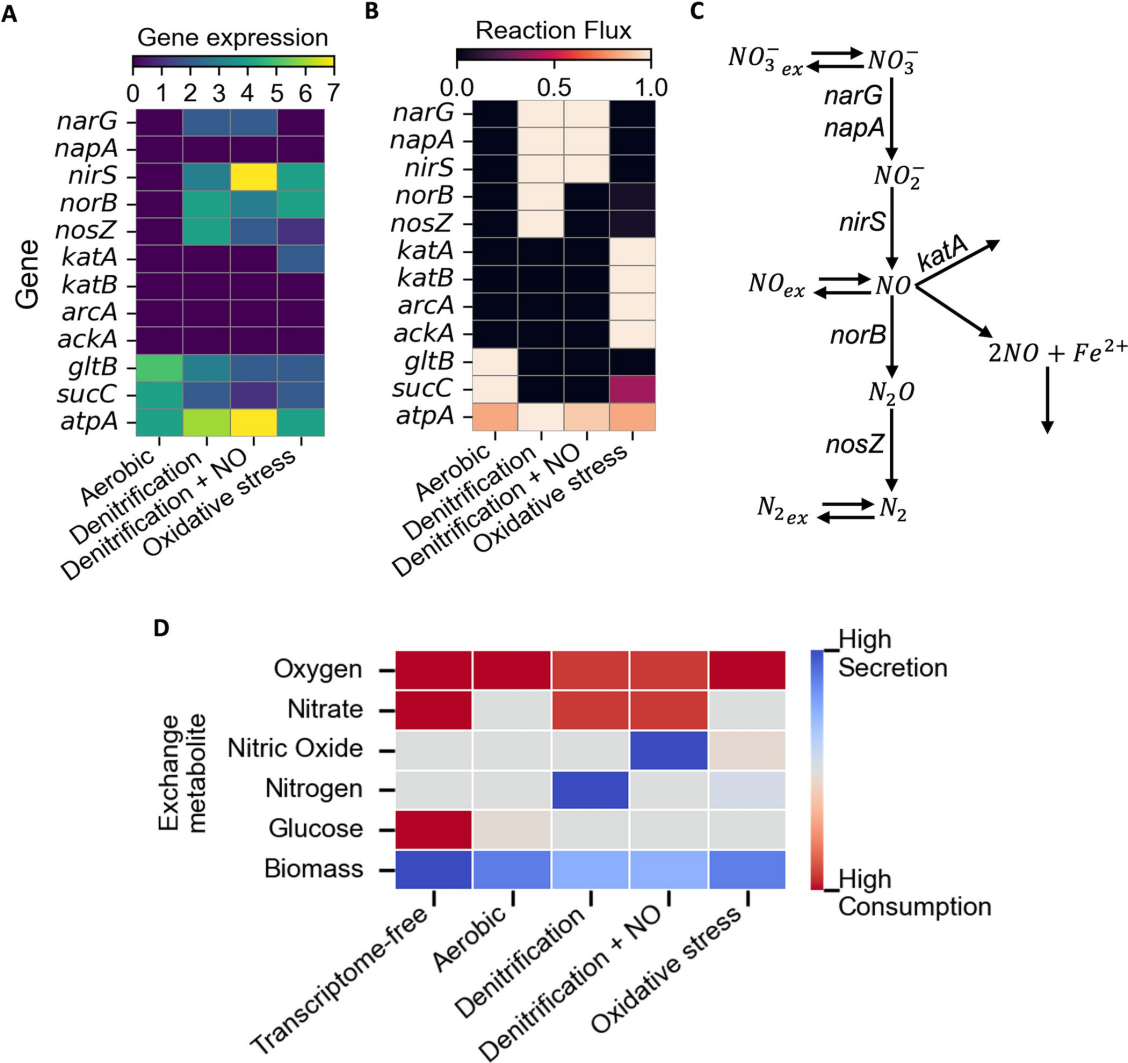
MiMICS incorporated four unique spatial-transcriptome-guided PA14 metabolic model states. (A) Heatmap of experimental gene expression values of each PA14 metabolic model state. (B) Heatmap of predicted flux values of gene-encoded reactions for each PA14 metabolic model state. Flux values reported are absolute flux values normalized to the maximum absolute flux value among metabolic model states. (C) PA14 GENRE denitrification and oxidative stress pathway updated from iPau21 GENRE with NO secretion pathway, NO iron cytotoxic reaction, and *katA*-encoded oxidative stress reaction. (D) Predicted fluxes of metabolites exchanged with the extracellular environment for a transcriptome-free PA14 metabolic model and the four transcriptome-guided PA14 metabolic model states. Fluxes reported are normalized to the maximum flux among metabolic models.

The aerobic respiration metabolic model, integrated with transcriptomic data of cells with high expression levels of *ccoN1*, which encodes the primary aerobic respiration oxidase [27], predicted high levels of oxygen uptake flux (Fig 2D). A denitrification PA14 metabolic model was generated from cells with high expression levels of genes encoding denitrification reductases, *narG*, *napA*, *nirS*, *norB*, and *nosZ* (Fig 2A), which convert nitrate to nitrogen to perform respiration in low oxygen environments [22] (complete PA14 GENRE denitrification pathway shown in Fig 2C). The denitrification metabolic model accurately predicted flux through the denitrification reductase reactions, resulting in characteristic nitrate uptake and nitrogen secretion fluxes (Fig 2B and 2D) [28]. A denitrification +NO secretion PA14 metabolic model was generated from cells with high *narG* and *nirS* expression, respectively encoding nitrate and nitrite reductase, but low *norB* expression, encoding NO reductase. This expression profile, considered to be limited by the *norB* expression, resulted in a metabolic model state with nitrate uptake and NO secretion flux (Fig 2D). When simulated by individual agents in MiMICS, this NO secretion rate was passed from the agent’s intracellular scale (GENRE) to extracellular scale (reaction-diffusion model), which is an essential step to produce the NO biofilm microenvironment that induces oxidative stress. Demonstrating the impact of transcriptomic data integration, the PA14 metabolic model without transcriptomic data integration, termed transcriptome-free in Fig 2D, predicted oxygen and nitrate uptake, but did not predict NO secretion denitrification metabolic processes (Fig 2D).

Lastly, an oxidative stress PA14 metabolic model was generated from cells with high expression of the gene *katA*, which encodes the antioxidant enzyme Catalase A [23] (Fig 2A). Accordingly, the PA14 GENRE was updated with NO cytotoxic and antioxidant Catalase A intracellular reactions [23]. Briefly, to mitigate cytotoxic NO binding to intracellular iron, Catalase A binds to NO, acting as a NO sink supplementary to NO degradation by NorB [23] (Fig 2C). Correspondingly, the oxidative stress metabolic model predicted NO was consumed (Fig 2D) and preferentially degraded by NorB for biomass synthesis before being diverted through the antioxidant Catalase A reaction to mitigate NO toxicity [23] (S2 Fig).

Constrained on replete SCFM, the aerobic PA14 metabolic model state predicted a biomass growth rate similar to an experimental growth rate of aqueous *P*. *aeruginosa* in SCFM [29] (S2 Fig). The denitrification +/-NO secretion PA14 metabolic models predicted the lowest biomass growth rates indicative of low oxygen conditions [30] (S2 Fig). In addition to the predicted NO secretion rates, the predicted oxygen, nitrate, and glucose exchange flux rates (Fig 2D) were provided as inputs to update the corresponding metabolite concentrations in the reaction-diffusion model. Due to the differences observed in *gltB* expression, which encodes a glucose binding protein, among metabolic model states (Figs 2A and Fig 2B), and previous reports relating denitrification metabolism and glucose uptake [31,32], glucose was chosen as the carbon source to simulate in the reaction-diffusion model.

### Mechanistic incorporation of transcriptome-guided PA14 metabolic models into MiMICS

MiMICS employs ABM rules for an agent to select a metabolic model state from a set of transcriptome-guided metabolic models input by the user. In this study, a combination of mechanistic ABM rules informed from literature and the spatial transcriptomic dataset were implemented into MiMICS (Table 1). *P*. *aeruginosa* has been observed to upregulate genes related to aerobic and denitrification metabolism in aerobic and deplete oxygen conditions, respectively [22]. In addition, in the presence of extracellular NO, *P*. *aeruginosa* increases expression of *katA*, encoding the antioxidant Catalase A [23]. Thus, to decide between the four PA14 metabolic model states, agents compared their local oxygen and NO concentrations to respective concentration thresholds, [O_2_]_t_ and [NO]_t_ (Table 1). The parameter value for [NO]_t_ was obtained from literature as the extracellular NO concentration that induced *katA* expression [23]. The parameter value for [O_2_]_t_ was fit to experimental outputs (S4 Fig). An oxic O_2_ threshold, 0.21 *mM*, had the smallest MiMICS model error, which suggests complete oxygen depletion was not essential to induce denitrification metabolism. Indeed, previous studies observed *P*. *aeruginosa* used denitrification in microaerobic conditions (~0.05 mM oxygen) as a possible supplementary or competitive respiration strategy to aerobic respiration [28]. The parameterized [O_2_]_t_ value in the oxic range can be expected because the height (~10 μm) of the ten-hour biofilm was not large enough to predict significant oxygen depletion [33]. In the case that lower oxygen concentrations were present in the biofilm, other mechanisms that deplete oxygen near the biofilm, such as an oxygen boundary layer [34] or oxygen consumption by the planktonic phase above the biofilm may be present. These mechanisms serve as updates to simulate metabolite transport in upcoming MiMICS versions. Altogether, MiMICS suggests an oxygen transition point emerged within the PA14 biofilm that induced denitrification metabolism, but oxygen depletion mechanisms in regions outside of the biofilm may have been present.

**Table 1:** Mechanistic ABM rules for *P*. *aeruginosa* agents to choose a PA14 metabolic model state. *P*. *aeruginosa* agents compared their local oxygen and nitric oxide concentration to respective metabolite thresholds. A stochastic parameter, R_n_, determined the agent’s decision between the denitrification +/- NO secretion metabolic models.

PA14 metabolic models	Oxygen^1^	Nitric oxide^2^	Stochastic parameter, R_n_
Aerobic	[O_2_] ≥ [O_2_] _t_	[NO] < [NO] _t_	No relation
Denitrification	[O_2_] < [O_2_] _t_	[NO] < [NO] _t_	R_n_ ≥ 0.06
Denitrification +NO	[O_2_] < [O_2_] _t_	[NO] < [NO] _t_	R_n_ < 0.06
Oxidative stress	No relation	[NO] ≥ [NO] _t_	No relation

^1^ [O_2_] _t_ = 0.21 *mM* was fit to experimental data

^2^ [NO] _t_ = 1 *μM* was informed from literature^22^

A stochastic parameter, R_n_, generated only for agents in the denitrification-inducing low oxygen and low NO condition, was used for agents to select between the two denitrification metabolic model states, one with and one without predicted NO secretion flux (Table 1). The R_n_ threshold was informed from experimental data, which suggested 6% of denitrification cells exhibited the *norB*-limiting gene expression profile associated with NO secretion (S2 Fig). This R_n_ parameter suggests cells stochastically expressed *nirS* or *norB*, which encode nitrite reductase and NO reductase respectively. Indeed, stochastic expression of denitrification genes has been observed in other bacterial species [35]. In addition, experimental observations of *P*. *aeruginosa* biofilms after ten hours showed high expression levels of *pilA* (S1 Fig), which encodes type IV pili protein (PilA) that facilitates surface motility and shapes biofilm structure [5,36]. As pili synthesis reactions were not in the current PA14 GENRE [37], simple surface motility ABM rules were incorporated to recapitulate the PA14 biofilm structure and total cell count (S3 Fig).

### Transcriptome-guided MiMICS predicted microscale metabolic heterogeneity and NO microenvironment in PA14 biofilm

**Fig 3:**
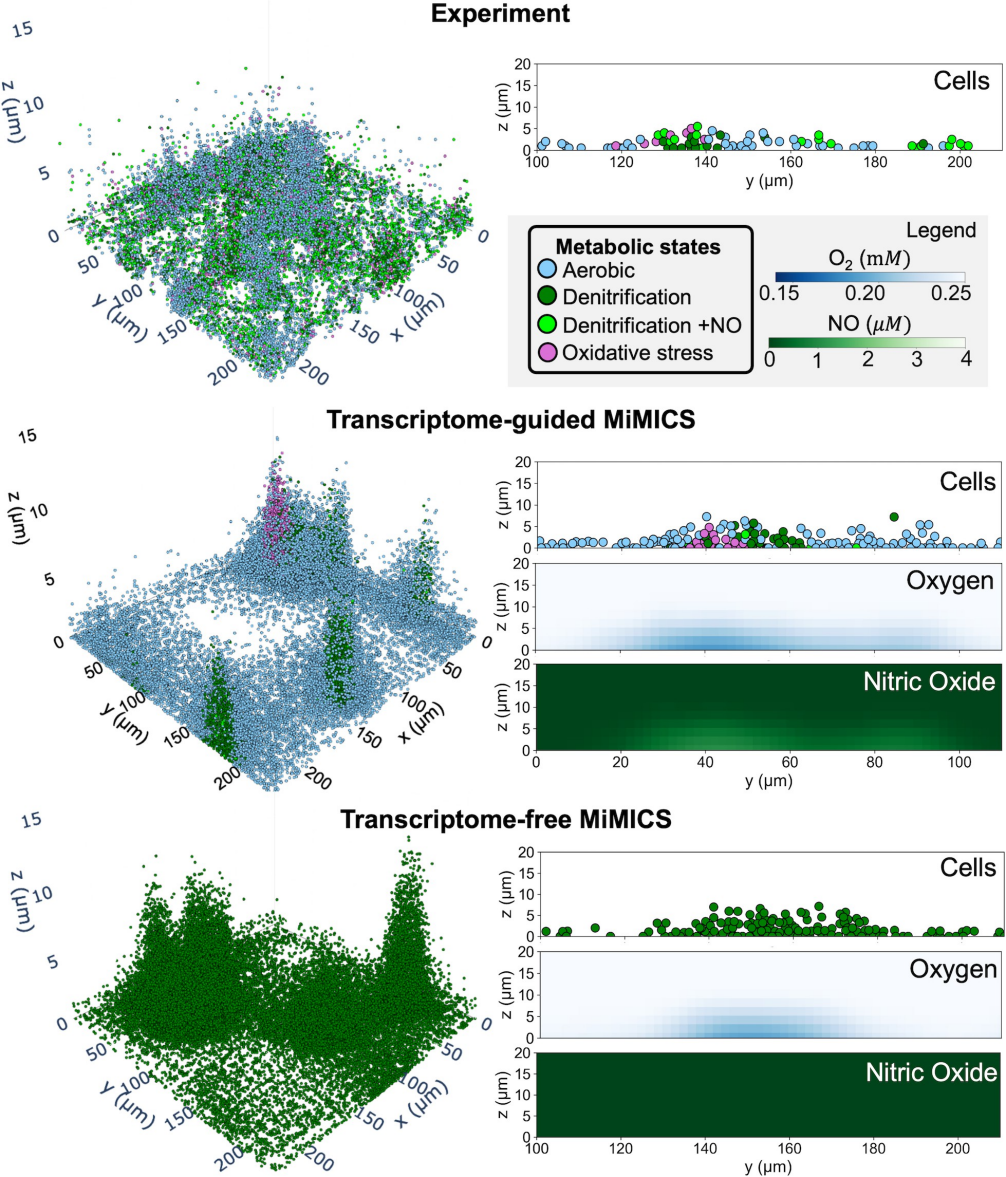
Transcriptome-guided MiMICS improved predictions of microscale metabolic heterogeneity and NO microenvironment in PA14 biofilm. Shown are representative 3D renderings of PA14 biofilms grown for ten-hours from the experiment, and transcriptome-free and transcriptome-guided MiMICS simulations. Cells are colored according to their metabolic state. Shown are 2D *yz* slices (x = 160 *μm*, 22 *μm*, 100 *μm* for experimental, transcriptome-guided MiMICS, and transcriptome-free MiMICS, respectively) of cell metabolic states, and predicted oxygen and NO concentrations. The x-values were chosen to compare similar biofilm colony structures across experimental and simulation conditions. Experimental data was reconstructed from Dar and co-workers.

MiMICS was simulated with the mechanistic ABM rules controlling agent execution of a transcriptome-guided PA14 metabolic model state (Table 1) (referred to as transcriptome-guided MiMICS simulation). Qualitatively, in comparison to the experiment, the transcriptome-guided MiMICS simulation accurately predicted microscale, spatially confined biofilm niches in which cells heterogeneously upregulated denitrification and oxidative stress metabolic processes (Fig 3). In the simulation, these niches were located near the center of the biofilm colony, where microaerobic and variable NO concentrations were predicted (Fig 3). Thus, MiMICS simulations suggested that variable NO signal concentrations within microaerobic biofilm regions resulted in a heterogeneous population of cells using denitrification and oxidative stress metabolism co-existing within the same microscale niche. Qualitatively, experimental PA14 biofilms observed greater spatial dispersion of cells expressing denitrification and oxidative stress genes (Figs 3 and S5), suggesting other mechanisms may regulate denitrification gene expression, such as stochastic expression of the denitrification gene *narG* encoding nitrate reductase [35].

To demonstrate the advantages of incorporating multiple transcriptome-guided metabolic models, MiMICS was simulated with the PA14 GENRE unconstrained by transcriptomic data (referred to as transcriptome-free MiMICS), which is the standard practice for current multi-scale metabolic frameworks [8,16,17]. Transcriptome-free MiMICS inaccurately predicted a homogeneous biofilm population with active flux through all intracellular denitrification metabolic reactions (Figs 3 and S5). In addition, transcriptome-free MiMICS did not predict extracellular NO in the biofilm microenvironment, resulting in agents lacking flux through the NO-induced oxidative stress reaction encoded by *katA* (Figs 3 and S5).

### Transcriptome-guided MiMICS improved prediction accuracy of heterogenous PA14 biofilm metabolism

**Fig 4:**
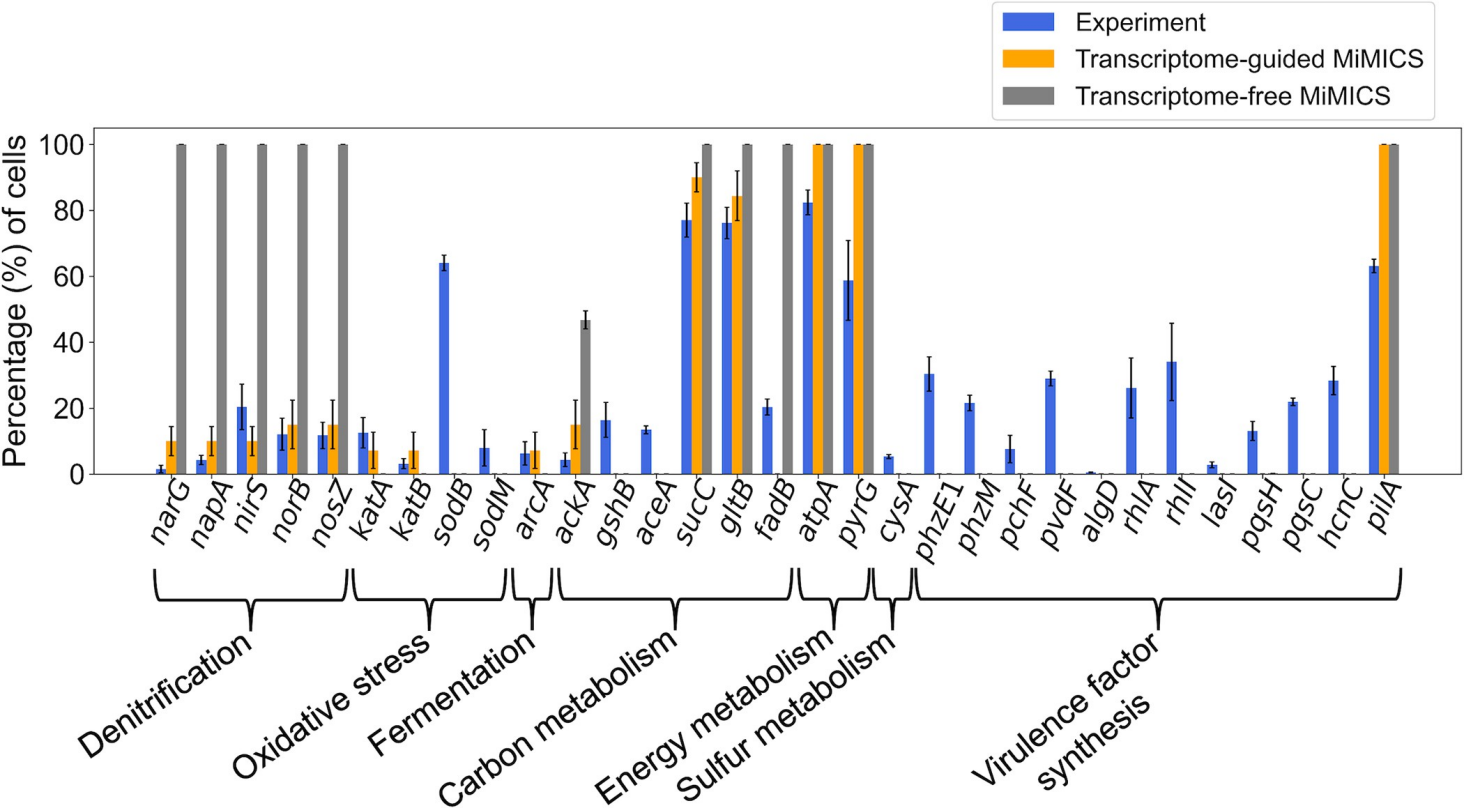
Transcriptome-guided MiMICS improved prediction accuracy of heterogeneous intracellular metabolism in PA14 biofilm compared to transcriptome-free MiMICS. From experimental data, plotted is the average percentage of total cells expressing a gene. From transcriptome-guided MiMICS and transcriptome-free MiMICS simulation data, plotted are the average percentage of total agents with active flux through the reaction encoded by a gene. Error bars represent one standard deviation from n = 7 experimental replicates and n = 50 simulation replicates. Experimental data provided by Dar and co-workers.

Quantitative validation of MiMICS was first performed by comparing the experimental percentage of total cells expressing a gene to the simulation’s percentage of total agents with active flux through metabolic reactions encoded by that respective gene (Fig 4). Compared to transcriptome-free MiMICS that predicted 100% of cells with active denitrification reaction flux, transcriptome-guided MiMICS accurately predicted reduced cell populations with active flux through denitrification *narG-*, *napA-*, *nirS-*, *norB-*, and *nosZ*-encoded reactions (Fig 4).

As a result of the ABM rules enforced in transcriptome-guided MiMICS (Table 1), a heterogenous agent distribution simulating the denitrification and oxidative stress metabolic model state emerged within the spatially variable NO biofilm microenvironment (Fig 3). Consequently, reflective of the intracellular metabolic model states (Fig 2B), transcriptome-guided MiMICS predicted a relatively low number of cells (10% of cells) utilizing *narG-* and *napA-*encoded reactions compared to the cell population utilizing *norB*- and *nosZ-*encoded reactions (15% of cells) (Fig 4). This MiMICS prediction agreed with the experimental results reporting that relatively few cells expressed *narG* and *napA*, 1.5% and 4.3% of cells, respectively, compared to cell populations with high *norB* and *nosZ* expression, 12.1% and 11.8% of cells, respectively (Fig 4).

Interestingly, transcriptome-guided MiMICS did not accurately predict larger cell populations expressing *nirS* (20.4% of cells in experiment, 10% in simulation) relative to cell populations expressing *narG*, *norB*, and *nosZ* (specific percentages reported above) (Fig 4). While NO has been observed to also upregulate *nirS* expression^32^, hypothesized to bind NO required for NO transport to NorB for degradation [38], the current PA14 GENRE does not include this NirS NO-binding reaction. Thus, the NO-induced oxidative stress metabolic model did not predict active *nirS-*encoded reaction flux (Fig 2B), likely accounting for MiMIC’s prediction discrepancy of a larger *nirS*-expressing cell populations (Fig 4).

Only the transcriptome-guided MiMICS simulation predicted a NO biofilm microenvironment (Figs 3 and S5), which was hypothesized to induce oxidative stress in nearby cells. Accordingly, only transcriptome-guided MiMICS simulations predicted a small population of cells (0% in transcriptome-free simulation, 7.2% in transcriptome-guided simulation, and 12.5% in experiment) with flux through the *katA-*encoded oxidative stress reaction (Fig 4).

In terms of carbon metabolism, both transcriptome-free and transcriptome-guided MiMICS correctly predicted a majority of cells with flux through a succinate dehydrogenase reaction used in the tricarboxylic acid (TCA) cycle, encoded by *sucC*, and a glucose transport reaction encoded by *gltB* (Fig 4). The transcriptome-guided MiMICS prediction of a large population of cells expressing *sucC* (90% of cells) and *gltB* (84% of cells) is a result of the flux through *sucC-* and *gltB-*encoded reactions that was predicted to be highest in the aerobic metabolic model state (Fig 2A and 2B), which most agents represented because a majority of agents were likely exposed to high oxygen. Transcriptome-guided MiMICS accurately reduced cell populations (46.8% in transcriptome-free simulation, 15.0% in transcriptome-guided simulation, and 4.4% in experiment) with active flux through an acetate kinase fermentation reaction, encoded by *ackA* [22] (Fig 4). Related to energy metabolism, both MiMICS transcriptome scenarios predicted 100% of cells with active flux through the ATP synthase reaction, encoded by *atpA*, and CTP synthase, encoded by *pyrG*, compared to experimental populations expressing *atpA* (82.4% of cells) and *pyrG* (58.8% of cells) (Fig 4).

Potential inaccuracies in either the -omics data-integration method or the MiMICS model components rendered the transcriptome-guided MiMICS incapable of predicting the relatively low cell proportions utilizing *gshB* and *aceA*, which encode glutathione synthetase and isocitrate lyase, respectively, or *cysA*, which encodes a sulfate transport protein (Fig 4). In addition, neither MiMICS transcriptome version captured heterogeneous expression of genes related to virulence factor synthesis, which was expected as virulence factor synthesis likely does not contribute to the biomass synthesis GENRE objective function used during transcriptome data integration [37]. Overall, compared to the standard practice of using a transcriptome-free multi-scale framework, transcriptome-guided MiMICS improved prediction accuracy of the heterogeneous cell distributions with upregulated denitrification and oxidative stress metabolism.

To investigate the impact of the ability for cellular agents to dynamically adopt different metabolic model states, a MiMICS scenario was run with cells that remained fixed in a randomly initialized metabolic state, called ‘Fixed metabolic state MiMICS’ (S9 Fig). Fixed metabolic state MiMICS predicted homogeneous metabolic state niches localized to the initial location of the respective metabolic state and limited spatial mixing of different metabolic states. In contrast, the dynamic switching ability in MiMICS promoted microscale niches of cells with a heterogeneous distribution of metabolic states localized to the biofilm center. This result confirms that dynamic metabolic state adaptation to the metabolic microenvironment is essential to recapitulate the confined microscale niches of heterogenous metabolic states observed in the experimental biofilm.

### MiMICS captured spatial relationships of intracellular metabolism in PA14 biofilm

**Fig 5:**
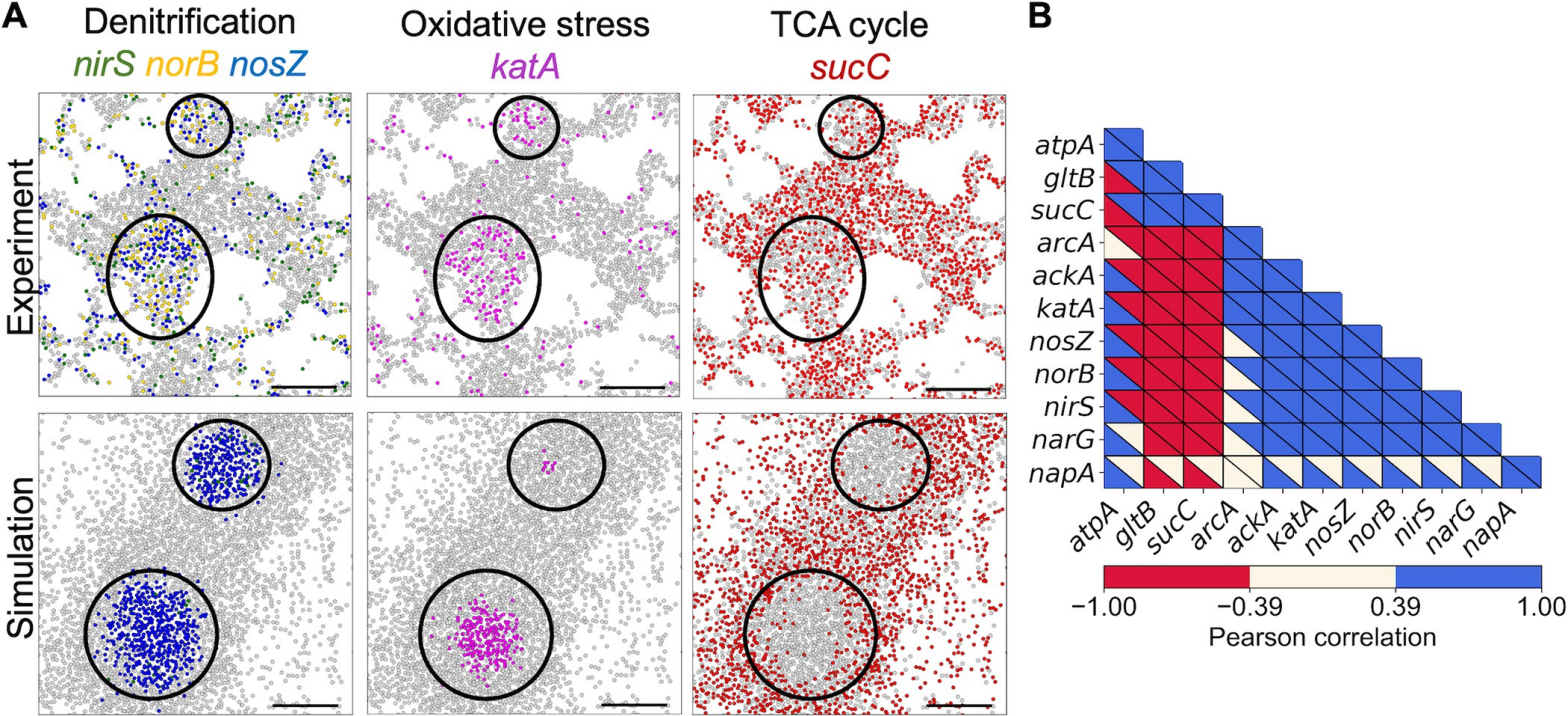
MiMICS captured spatial relationships of intracellular metabolism in PA14 biofilm. (A) Representative *xy* projections of PA14 biofilms from experiments and transcriptome-guided MiMICS simulations. Cells plotted are located near the z = 0 *μm* surface. Colored cells have high expression of the gene listed (experiment) or high reaction flux encoded by the gene listed (simulation). Circled areas highlight regions of interest where there is an anticorrelation between TCA cycle metabolism with denitrification and oxidative stress metabolism. Scale bar represents 20 *μm*. (B) Neighborhood gene spatial correlation comparison between experiment and transcriptome-guided MiMICS simulation. Spatial correlation between gene pairs was assessed by a Pearson correlation, where +1 and -1 value correspond to a strong positive and strong negative spatial correlation, respectively. The experimental and simulation Pearson correlation values are plotted in the upper right and lower left triangle of each square, respectively. Simulation Pearson correlation values were determined from 50 simulation replicates. Experimental data was reconstructed from Dar and co-workers.

To quantitatively evaluate the validity of transcriptome-guided MiMICS predictions in space, a bulk neighborhood spatial correlation analysis between genes was performed, similar to the analysis of experimental data by Dar and co-workers [5] (Fig 5B). Genes were compared which had both positive experimental expression values and simulated active flux values of reactions encoded by the respective gene. Shown in Fig 5B, MiMICS accurately predicted the spatial correlation for 33 gene pairs, and incorrectly predicted the spatial correlation for 22 gene pairs. Specifically, MiMICS accurately predicted that denitrification genes (i.e. *narG*, *nirS*, *norB*, *nosZ)* were positively correlated with one another, and positively correlated with the oxidative stress gene *katA* (Fig 5A and 5B). Conversely, MiMICS predicted *napA*, which encodes nitrate reductase, was positively correlated with the remaining denitrification genes *narG*, *nirS*, *norB*, *and nosZ*. However, this correlation was not observed in the experiment. Upon closer inspection, MiMICS predicted patches in the biofilm where neighboring cells utilized *napA-*, *nirS-*, *norB-*, *and nosZ*-encoded reactions (S6 Fig). In contrast, cells expressing *napA* in the experiment were more dispersed compared to more spatially confined biofilm regions of cells expressing *nirS*, *norB*, and *nosZ* (S6 Fig). MiMICS prediction discrepancies in the *napA* spatial correlations suggest alternate mechanisms exclusively modulate *napA* expression, such as extracellular phenazine secretion [39]. Indeed, phenazine synthesis genes *phzE1* and *phzM* were expressed in the ten-hour PA14 biofilm (Fig 4). In addition, MiMICS accurately predicted denitrification and oxidative stress genes (i.e. *narG*, *nirS*, *norB*, *nosZ*, *katA)* were anticorrelated with the carbon metabolism genes *gltB* and *sucC* (Fig 5A and 5B). One aspect MiMICS did not accurately predict was the spatial correlation of *atpA*, encoding ATP synthase, with all other genes (Fig 5B), which motivates improvements in future transcriptome-guided MiMICS simulations.

### MiMICS revealed functional dynamics of denitrification and oxidative stress metabolism in PA14 biofilm

This spatial transcriptomics experiment required biofilm fixation, preventing *in-situ* dynamic monitoring of gene expression within the biofilm sample. Thus, this spatial transcriptomic method was unable to characterize the temporal events that underline the PA14 biofilm gene expression distributions and spatial patterns. In contrast, transcriptome-guided MiMICS dynamic simulations were used to monitor and quantify spatiotemporal shifts of PA14 biofilm metabolism connected to the metabolite microenvironment.

**Fig 6:**
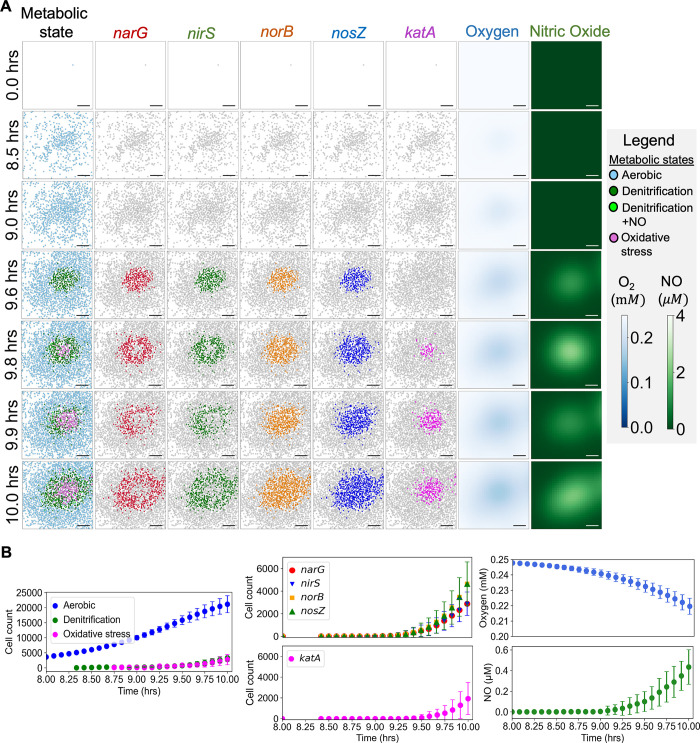
MiMICS revealed the microscale dynamics of denitrification and oxidative stress intracellular metabolism, and extracellular nitric oxide (NO) in the PA14 biofilm. (A) Representative *xy* projections at a z = 0 μm slice of a simulated PA14 biofilm colony developing over time. Agents are colored according to their assigned metabolic model state. Agents are also colored according to active flux through reactions encoded by denitrification genes (*narG*, *nirS*, *norB*, and *nosZ)* and encoded by the oxidative stress gene *katA*. Plotted are the corresponding *xy* projections of oxygen and NO concentrations at the z = 0 μm slice. Scale bar represents 10 *μm*. (B) Quantified dynamics of the average number of agent’s assigned a metabolic model state, and the average number of agents with active reactions flux encoded by denitrification and oxidative stress genes. Dynamics of the average extracellular oxygen and NO in the biofilm are reported. Error bars represent one standard deviation from 50 simulation replicates.

Agents were randomly initialized at t = 0hrs, nucleating the simulated PA14 biofilm which was grown over a period of ten hours (Fig 6). After approximately nine hours of simulated PA14 biofilm growth, agents exposed to the emergent microaerobic biofilm environment (i.e. local oxygen below the [O_2_]_t_ value) switched from the aerobic to denitrification metabolic model state (Fig 6A and 6B). In accordance with the aerobic and denitrification metabolic model states (Fig 2A), cell populations with active flux through *narG-*, *nirS-*, *norB-*, and *nosZ*-encoded denitrification reactions increased over time (Fig 6A and 6B). The stochastically-chosen agents that executed the *norB*-limiting denitrification metabolic model (Table 1) secreted NO, generating the NO biofilm microenvironment (Fig 6A). The maximum level of NO, ~4 *μ*M, below toxic levels [23], was localized in the biofilm center (Fig 6A). At ten hours, agents that sensed local extracellular NO above the [NO]_t_ value switched to the oxidative stress metabolic model state, resulting in increased cell populations with flux in the oxidative stress reaction encoded by *katA* (Fig 6).

Due to the ABM rules that were enforced (Table 1), a heterogeneous distribution of cells using the denitrification and oxidative stress metabolic models emerged within microaerobic and spatially variable NO biofilm microenvironments. Only the denitrification +/-NO metabolic model states predicted active flux through *narG*- and *nirS*-encoded intracellular reactions, which are upstream of NO synthesis and required for NO secretion (Figs 2B and 6A). Conversely, denitrification and oxidative stress metabolic model states predicted active flux through *norB*- and *nosZ*-encoded intracellular reactions, which are downstream of NO synthesis and used for NO degradation (Figs 2B and 6A). As a result of these intracellular differences among metabolic model states, fewer cells in the biofilm were predicted to use *narG*- and *nirS*-encoded reactions compared to cells using *norB*- and *nosZ*-reactions (Fig 6B).

Relating these MiMICS dynamic predictions to possible biological functions, previous studies similarly observed cells downregulated *narG* expression and upregulated expression of *nirS* and *norB*, the latter encoding a NO degradation reductase, in presence of NO [40,41]. This NO-induced regulation of different genes in the denitrification pathway was hypothesized to arrest NO synthesis, promote NO degradation, and maintain extracellular NO within non-toxic concentrations [40,41]. Functioning similarly, MiMICS predicted NO signaled agents to reduce flux through *narG* and *nirS*-encoded NO synthesis reactions, which decreased NO synthesis and secretion (Fig 6). Due the decreased NO secretion, and extracellular NO gradients dissipated at 9.9 hours in the biofilm colony shown in Fig 6A. In contrast, possibly to promote degradation of extracellular NO, agent populations with active flux through *norB-* and *nosZ-*encoded NO degradation reactions continued to increase (Fig 6).

Next, possibly due to the reduced NO concentrations and expanding microaerobic biofilm regions, agents switched to a denitrification +/-NO secretion metabolic model state, again promoting a NO-rich biofilm microenvironment at 10 hours in the biofilm colony in Fig 6A. Thus, MiMICS predicted an oscillating, non-toxic NO biofilm microenvironment (Fig 6). This prediction is supported by previous studies that have measured temporal oscillations in extracellular NO in low oxygen *P*. *aeruginosa* cultures performing denitrification [42,43]. Furthermore, temporal oscillations in expression of mRNA encoding denitrification reductases has been observed in *E*. *coli* [44]. In MiMICS, the predicted oscillations in extracellular NO were a result of cellular agents differentially regulating intracellular reactions in the denitrification pathway in response to extracellular oxygen and NO, which may function to maintain a non-toxic NO biofilm microenvironment. Future simulations can systematically perturb genes and parameters in MiMICS to test their effect on predicted outcomes, such as NO metabolism in the biofilm. For example, *in silico* gene knock-outs, such as Δ*nirS* that increases NO secretion, or varied extracellular nutrient concentrations may be screened for increased NO-induced *P*. *aeruginosa* cell death as a potential therapeutic strategy. Simulation readouts, such as biofilm structures, gene expression, or number of live/dead cells, can be quantified and compared to the experiment.

### MiMICS computational performance

Performing GENRE simulations for individual cellular agents is advantageous to predict emergent metabolic heterogeneity within a multicellular system but can be computationally expensive. Execution of one MiMICS simulation time step on one central processing unit (CPU) resulted in a runtime on the order of 10 minutes for 10,000 cellular agents. To improve this MiMICS runtime, GENRE simulations for each individual agent were divided across multiple CPUs using parallel computing, implemented with Python Multiprocessing. This parallel computing strategy reduced MiMICS runtime by an order of magnitude for 10,000 cellular agents (S7 Fig). This computational performance is similar to simulation runtimes reported by the BacArena framework [17] for 1000 agents, where MiMICS is the first framework to report efficient runtimes for up to 10,000 single-cell agents. The number of metabolic model states input into MiMICS did not considerably impact MiMICS runtime (S7 Fig).

## Conclusion

In this work, a novel multi-scale computational framework, MiMICS, was developed and presented. MiMICS is an open-source computational framework that interfaces Python-based metabolic models with the established Java-based Hybrid Automata Library agent-based model and reaction-diffusion model platform. MiMICS is extendable for users to input multiple -omics data-integrated metabolic model states that yield different metabolic parameter values that are then passed to the extracellular models.

Used as a test case to demonstrate the framework’s utility, MiMICS simulated the connected, multi-scale metabolic processes controlling microscale metabolic heterogeneity observed in a *P*. *aeruginosa* PA14 biofilm. MiMICS applied mechanistic ABM rules for *P*. *aeruginosa* agents to choose a spatial transcriptomic-guided PA14 metabolic model state. As a result, MiMICS predicted microscale niches where cells heterogeneously upregulated denitrification and oxidative stress metabolism mapped to a microaerobic and variable NO microenvironment. Transcriptome-guided MiMICS revealed the spatial dynamics of cells heterogeneously regulating reactions in the denitrification pathway, possibly to maintain a non-toxic NO biofilm microenvironment. Future MiMICS perturbations could seek to promote NO-induced cell death to inform potential PA14 biofilm therapeutic strategies. While not supported by the experimental dataset that was measured at widely spaced time points, NO-induced biofilm dispersal [45] could be added to the MiMICS mechanistic rule set to simulate dynamic biofilm restructuring.

In this work, the single-cell spatial transcriptomics dataset was used to calibrate the metabolic model component of MiMICS, and MiMICS predictions were tested with metrics at the multicellular level (e.g. gene neighborhood Pearson correlation). To calibrate the metabolic model with -omics data independent of the test dataset, future MiMICS applications could integrate global or single-cell transcriptomics data that was measured independently in relevant metabolic conditions (e.g. varying oxygen concentrations). Future work could also extend MiMICS to explore other mechanisms, such as quorum-sensing signals, that regulate heterogeneous metabolism in single- or multi-species biofilms. Overall, we believe MiMICS is a valuable computational tool to integrate -omics data and elucidate mechanisms that control metabolic heterogeneity, which can promote virulence and antibiotic tolerance in microbial biofilms.

## Methods

### Genome-scale network reconstruction (GENRE)

*Pseudomonas aeruginosa* PA14 metabolism was simulated with a published *P*. *aeruginosa* PA14 genome-scale metabolic network reconstruction (GENRE) (iPau21) [12,37]. All GENRE simulations were performed in Synthetic Cystic Fibrosis Sputum Medium (SCFM). A few reactions were updated in the PA14 GENRE to represent the denitrification, fermentation, nitric oxide secretion, and oxidative stress metabolic processes observed in experiments. PA14 GENRE simulations in anaerobic + nitrate were initially infeasible (S8 Fig), which did not reflect expected denitrification biomass synthesis processes [22]. In anaerobic SCFM conditions, ubiquinol-9 (UQ_9_), a preferred respiration cofactor required in the PA14 GENRE biomass objective function [37], synthesis was found to be blocked which prevented biomass growth (S8 Fig). Three oxygen-independent hydroxylation reactions of UQ_9_ precursor metabolites were added with H_2_O as the reactant in the place of oxygen, a hypothesized oxygen-independent reaction [46,47,48]. Oxygen-dependent UQ_9_ synthesis reactions were set to be irreversible to prevent the generation of intracellular oxygen. To promote feasible L-arginine fermentation, reactions encoded by *arcB* and *argH* were set as reversible and irreversible, respectively, in agreement with the expected arginine deiminase pathway [49]. These updates to the PA14 GENRE promoted feasible biomass growth rates and flux through the respective reactions in anaerobic conditions (S8 Fig). Exchange and transport reactions were added for the denitrification intermediates nitric oxide (NO) and nitrous oxide (N_2_O). NO toxicity was represented as a sink reaction for NO and iron [23] and an oxidative stress KatA reaction was represented as a sink reaction for NO [23]. Of note, when cells were exposed to an extracellular NO microenvironment in MiMICS, NO uptake was enforced by fixing the GENRE upper bound for NO exchange at a negative flux value, which was calculated from the cell’s local extracellular NO concentration (Eq 1).

In MiMICS, each agent’s PA14 metabolic model was optimized for a biomass synthesis objective function using flux-balance analysis (FBA) [9]. Each agent’s metabolic model exchange reaction bounds were constrained by the agent’s local oxygen, nitrate, glucose, and nitric oxide concentrations. The agent’s local patch metabolite concentrations and metabolic model lower bound of exchange reaction flux were converted with the following equation:

$$f_{M}=\frac{c_{M}*v_{patch}}{{dt}_{ex}*b}$$ (1)

Where *f*_*M*_ is the metabolite flux that set the metabolic model exchange flux (mmol/(gdWt *hr), *c*_*M*_ is the agent’s local patch metabolite concentration (mM), *v*_*patch*_ is the patch volume unoccupied by the bacteria (L), *dt*_*ex*_ is the metabolite uptake time step (estimated as 0.05s in Table 2), and *b* is the agent biomass (g). *v*_*patch*_ was assumed as 1e^-16^ L.

**Table 2:** MiMICS parameters and estimated time scales.

Description	Parameter	Value	Unit	Reference
World dimensions	*V*_*world*_	230 x 230 x 40	*μm* ^3^	[5]
Patch dimensions	*V*_*patch*_	2 x 2 x 2	*μm* ^3^	[50]
Metabolite diffusion time for gaseous metabolites	*dt*_*D*_*gas*_	0.0025	seconds	Estimated
Metabolite diffusion time for carbon metabolites	*dt*_*D_carbon*_	0.01	seconds	Estimated
Metabolite uptake time	*dt*_*ex*_	0.05	seconds	[2]
Time step for surface motility via pili	*dt*_*mot*_	5	minutes	[51]
Time delay for mRNA transcription	*dt*_*mRNA*_	10	minutes	[52]
*P*. *aeruginosa* biomass growth time step	*dt*_*growth*_	0.083	hours	Estimated
Simulation time step	*dt*_*sim*_	5	minutes	Estimated

### Construction of transcriptome-guided PA14 metabolic models

The PA14 GENRE was integrated with a published spatial transcriptomics PA14 biofilm dataset [5] using the established RIPTiDe algorithm [11]. The dataset consisted of the mRNA expression of ~292,000 PA14 biofilm cells grown in SCFM, and fixed at either 10hr (n = 7) or 35hr (n = 3) growth time points [5]. For data integration into the PA14 GENRE, gene expression values were only used for the 47 of the 105 genes measured in the experiment that also encoded reactions in the PA14 GENRE (S1 Table). For data integration into the PA14 GENRE, lower bounds for exchange reactions were constrained according to SCFM concentrations (S2 Table), converted using Eq 1. In addition, upper bounds for exchange reactions were set to +1000 to allow for unconstrained production of exchange metabolites.

A UMAP Leiden clustering analysis (scanpy v.1.7.0), data preparation and parameters described elsewhere [5], identified distinct gene expression cellular states within the spatial transcriptomic PA14 biofilm dataset (S1 Fig). Metabolic states for each cluster were assigned based on the highest ranked genes within the cluster. Median gene expression was extracted for the top 9 clusters, which captured 91% of biofilm cells, that were integrated into the PA14 GENRE using RIPTiDe [11] to generate 9 UMAP-identified PA14 metabolic model states. A fractional growth rate of 0.7 was used in RIPTiDe. Reactions not shared by all UMAP-identified metabolic model states, termed non-consensus reactions, and predicted exchange metabolite fluxes were compared and grouped based on similarity to identify unique 10-hour and 35-five PA14 metabolic state models (S1 Fig). Due to the wide diversity of metabolic states between the 10hr and 35hr time points, the initial scope of this study was on metabolic states emerging within PA14 biofilms grown for 10 hours. This UMAP-informed method generated the 10hr biofilm aerobic PA14 metabolic model state that was input into MiMICS.

A manual selection method was used to extract gene expression values of denitrification and oxidative stress metabolic subpopulations that were not identified in the UMAP Leiden clustering analysis. The ~22,000 denitrification cells classified in the clustering analysis were categorized into having high expression of *narG* and/or *napA* (S2 Fig). The *narG*- and *napA*-expressing denitrification-classified cells were further classified into possessing a non-limiting or limiting denitrification pathway (S2 Fig). Cells with a non-limiting denitrification pathway did not possess a rate limiting denitrification transcriptional step (i.e. expression of *narG < = nirS* < = *norB < = nosZ*). Cells categorized with limited denitrification metabolism had expression of a gene encoding a denitrification reductase that was larger than the expression value of the gene encoding the subsequent denitrification reductase gene. From cells classified with non-limiting and limiting denitrification metabolism, the median gene expression was extracted and integrated into the PA14 GENRE using RIPTiDe. The fraction of the optimal biomass was set as 0.55 used in RIPTiDe for these denitrification subpopulations [30]. The method generated four PA14 denitrification metabolic model states: no limiting denitrification step, *nirS*-limiting, *norB*-limiting, and *nosZ*-limiting. Predicted exchange fluxes of each of these denitrification limiting metabolic model states are shown in S2 Fig. This method generated the denitrification +/- NO secretion metabolic model states that were input into MiMICS.

An oxidative stress PA14 metabolic model state was generated by integrating the PA14 GENRE with median gene expression values extracted from the ~22,000 denitrification-classified cells with high *katA* expression. To simulate the exposure of an NO-induced oxidative stress environment, during transcriptome integration into the PA14 GENRE, the upper bound for the NO exchange reaction was set to a negative NO flux value (converted from 20 *μ*M NO) to enforce NO uptake. The fractional optimal biomass was 0.7 used in RIPTiDe. This method generated the oxidative stress PA14 metabolic model that was input into MiMICS.

### Metabolite reaction-diffusion model

Metabolite concentrations were simulated with the built-in HAL partial differential equation (PDE) alternating direction implicit (ADI) method solver. In HAL, the metabolite grids were defined as 3-dimensional 230 *μm* x 230 *μm* x 40 *μm* grids divided into 2 *μm* x 2 *μm* x 2 *μm* patches (Table 2). Metabolite grids were constructed for extracellular oxygen, nitrate, nitric oxide, and glucose.

Metabolite concentrations were initialized in each patch based on a Synthetic Cystic Fibrosis Sputum Medium (SCFM) recipe (S2 Table ) [29]. The initial condition for the metabolite PDEs was assumed to be spatially uniform:

$$c(x,y,z,t=0)=c_{initial}$$

where *c*_*initial*_ was the initial metabolite concentration (mM).

Before metabolite diffusion simulations, a metabolite reaction term was discretely applied at each agent’s respective grid location, representing the local metabolite consumption or production by that agent. The reaction term consisted of first converting the agent’s metabolic flux, predicted by the agent’s metabolic model, to a concentration (Eq 1), which was then passed to update the metabolite concentration at the agent’s grid location (Eq 2).

$$c_{f}=c_{0}+\Delta c$$ (2)

where *c*_*f*_ is the updated metabolite concentration (mM) at the agent’s location, *c*_0_ is the initial metabolite concentration (mM) at the agent’s location, and Δ*c* is the agent’s metabolic model predicted metabolic flux converted to a concentration (mM). With a consideration towards the thousands of agents, each with multiple predicted metabolic reaction rates, this discretely applied metabolite reaction rate approach was used to improve computational efficiency.

Next, diffusion was simulated using HAL’s ADI diffusion equation solver method. The governing equation for metabolite diffusion was as follows:

$$\frac{\partial c}{\partial t}=D\left(\frac{\partial^{2}c}{\partial x^{2}}+\frac{\partial^{2}c}{\partial y^{2}}+\frac{\partial^{2}c}{\partial z^{2}}\right)$$ (3)

where *c* is the metabolite concentration (mM), *t* is time (s), *x* is the x-dimension, *y* is y-dimension, *z* is the z-dimension, and *D* is the metabolite diffusion coefficient (cm^2^/s).

No flux boundary conditions were applied in the z-dimension:

$${\frac{\partial c}{\partial z}|}_{z=0}={\frac{\partial c}{\partial z}|}_{z=L}=0$$

where *z* = 0 was at the glass slide and *z* = L was at the air-aqueous interface.

Periodic boundary conditions in the x- and y-dimensions were applied. The biofilm region was defined as *R*_*biofilm*_. Regions outside of *R*_*biofilm*_ assumed constant metabolite concentrations. This assumption was attributed to higher metabolite diffusion coefficients occurring in the aqueous phase compared to metabolite diffusion coefficients the biofilm. In addition, the aqueous nutrient-rich media was refreshed every four hours in the experiment [5], so constant nutrient concentrations in the aqueous phase were assumed. To apply this assumption, for every metabolite diffusion step, the metabolite concentrations were set to the initial concentration in regions outside of the biofilm:

$$c(x,y,z,t)=c_{initial}\;if\;(x,y,z)\;is\;not\;in\;R_{biofilm}$$

Aqueous diffusion coefficients were multiplied by 0.6 for light gases and 0.25 for organic solutes to calculate the diffusion coefficient in a biofilm (Table 3) [53]. Biofilm diffusion coefficients were scaled according the recommended HAL equation for PDE stable solutions [24]:

$$D_{scaled}=\frac{D*{dt}_{D}}{{dl}^{2}}$$ (4)

where *D* is the metabolite diffusion coefficient (cm^2^/s), *dt*_*D*_ is the diffusion time step (seconds), and *dl* is the patch length (cm) (2 *μm* in Table 2). Gaseous and carbon metabolite diffusion coefficients were calculated according to the respective metabolite diffusion time scales, *dt*_*D_gas*_ and *dt*_*D_carbon*_, in Table 2.

**Table 3:** Metabolite diffusion coefficient values used to solve metabolite PDEs.

Description	Parameter	Value	Unit	Scaled value	Reference
Oxygen diffusion coefficient in biofilm	*D*_*o2*_	1.10 x 10^−5^	*cm* ^*2*^ */s*	0.72	[53]
Nitric oxide diffusion coefficient in biofilm	*D*_*NO*_	2.21 x 10^−5^	*cm* ^*2*^ */s*	0.78	[54]
Nitrate diffusion coefficient in biofilm	*D*_*NO3-*_	1.10 x 10^−5^	*cm* ^*2*^ */s*	0.7	[21]
Glucose diffusion coefficient in biofilm	*D*_*Gl*_	1.70 x 10^−6^	*cm* ^*2*^ */s*	0.4	[53]

### Agent-based model

The agent-based model (ABM) was constructed in the established Java-based platform Hybrid Automata Library (HAL) [24]. The ABM world was defined as a 3-dimensional 230 *μm* x 230 *μm* x 40 *μm* grid divided into 2 *μm* x 2 *μm* x 2 *μm* patches. Periodic boundary conditions were set in the x and y-directions. An individual agent represented an 8 *μm*^3^ single-cell *P*. *aeruginosa* bacterium. The agent class was built using HAL’s off-lattice SphericalAgent3D agent class. Each agent was assigned a unique index number. Bacteria agents were randomly initialized at z = 0 *μm* with a random biomass in the range of 1e^-12^ to 2e^-12^ g and a random directional angle. Biomass growth was calculated with an exponential growth rate law [5]:

$$b=b_{0}\;\exp\left[\mu *{dt}_{growth}\right]$$ (5)

Where *b* is the updated agent biomass in grams, *b*_0_ is the initial agent biomass in grams, *μ* is the growth rate informed from the agent’s optimized metabolic model (*hr*^*-1*^) and *dt*_*growth*_ is the growth time step (*hr*) (Table 2). If an agent biomass grew above the maximum biomass threshold (Table 4), cell division occurred, and a new daughter agent was placed in a neighboring patch of the mother agent. The mother agent biomass was randomly divided between the mother and daughter agent. The daughter agent’s directional angle was set to within 10 degrees of the mother agent’s angle. The daughter agent’s metabolic state was set as the mother agent’s metabolic state. An agent became inactive, representing cell death, in the agent-based model simulation if the predicted growth rate was 0.0 hr^-1^.

**Table 4:** Parameter values used for bacteria agents.

Description	Parameter	Value	Unit	Reference
Maximum biomass threshold		2e-12	g	[56]
Swarming speed		10	nm/s	[51]
Force scaler		0.5	N/m	[57]
Friction coefficient		0.2		[58]
Oxygen threshold to induce denitrification state	[O_2_]_t_	0.21	*mM*	Fit to data
NO threshold to induce oxidative stress state	[NO]_t_	1	*μM*	[23]
Growth lag phase		1.5	hours	[5]
Initial cell number		5	cells	Estimated

Simple ABM rules for *P*. *aeruginosa* surface motility were implemented based off previous observations of physical cell-cell interactions and collective swarming movement of piliated *P*. *aeruginosa* [36,55]. Pili surface motility speed was previously reported as 10 nm/s [51]. Thus, for a 5 minute simulation time step, one piliated bacteria agent was estimated to move 3 *μm* or 1.5 patches. For each five-minute simulation time step, the surface motility ABM function was performed once for each piliated bacteria agent. To determine the agent’s direction of movement, an agent on the surface (z = 0 *μm*) with at least one unoccupied and occupied neighbor patch set its orientation angle equivalent to a random neighbor agent. Agents moved to the new location if there were fewer than 20 cells in the surrounding region of the new location. For motility simulations, all bacteria agents in the ten-hour biofilm growth period were assumed to express *pilA* (S1 Fig) and perform the surface motility ABM function. For non-motile simulations, the motility ABM function was removed for all agents. In addition, to prevent agent overlap, the built-in HAL cell mechanics algorithm was executed for each agent using force scalar and friction coefficient parameters listed in Table 3.

Each agent was assigned their own metabolic model to simulate metabolism. Four PA14 metabolic model states were available for agents to choose from, specifically, the aerobic, denitrification, denitrification +NO secretion, and oxidative stress metabolic model states. Agents decided their metabolic model state by comparing their local oxygen and NO concentrations to the respective metabolite threshold values, [O_2_]_t_ and [NO]_t_ (Tables 1 and 4). For agents exposed to low oxygen and low NO environments, a stochastic parameter, R_n_, generated a number between 0 and 1. These cells decided between a denitrification with or without NO secretion metabolic model state by comparing their R_n_ value to a threshold value, 0.06, which was informed from the experimental percentage of denitrification cells with a *norB*-limiting expression profile. After agents checked these conditions, the agents were given a metabolic state assignment in the ABM, represented by an integer value. Used to assign the correct metabolic model state to each agent in Python, this metabolic model state integer value corresponded to the order in a list of metabolic model states input into MiMICS.

A biologically-relevant 10 minute time delay, *dt*_*mRNA*_, for agent’s to switch to a new metabolic model state upon sensing a new environmental cue was implemented, corresponding to the 10 minute delay for synthesis and detection of mRNA transcription upon a cell sensing a new environmental metabolite cue [52] (Table 2). Thus, when an agent encountered a new metabolite cue that induced a metabolic model state switch, the time the bacteria agent occupied that new metabolite environment was recorded. Once this recorded time exceeded 10 minutes [52], the bacteria agent was assigned the new metabolic model state. For dividing agents, this recorded time was equally split between the mother and daughter agents, representing observations of detected mRNA amounts in dividing cells [52].

## Coupled model components in MiMICS

For each simulation time step, *dt*_*sim*_, MiMICS executed each model component in the following order: 1) perform agent functions in the ABM, 2) constrain and optimize agent metabolic models, and 3) solve metabolite reaction-diffusion PDEs. A bacterial growth time scale was estimated as 30 minutes based on the doubling time of *P*. *aeruginosa* in SCFM [29]. Bacterial growth was determined as the rate-limiting biological process. Due to the expected stochasticity of bacteria agent division events, one MiMICS simulation time step, *dt*_*sim*_, was set as 5 minutes.

At the beginning of the time step, agent functions were performed in the ABM in order of agent death, division, metabolic state assignment, motility, and agent mechanics. Next, each agent’s biomass, index value, metabolic state value, and the agent’s local metabolite concentrations (i.e. oxygen, nitrate, NO, glucose) was passed from the ABM (Java) to the GENRE (Python). The metabolite concentrations were converted to fluxes using Eq 1 to constrain the agent’s metabolic model exchange fluxes. In transcriptome-guided MiMICS simulations, the agent’s metabolic state assignment number was used for agents to select a metabolic model state from the four PA14 metabolic model states. In transcriptome-free MiMCIS simulations, all agents simulated metabolism with PA14 GENRE constrained on SCFM, but not integrated with transcriptomic data.

Each agent’s GENRE was optimized for a biomass growth rate that was used to calculate (Eq 5) and update the agent biomass in the ABM. In addition, each agent’s optimized metabolic model predicted metabolite secretion and consumption fluxes. The oxygen, nitrate, NO, glucose exchange fluxes predicted by each agent’s metabolic model were converted to concentrations (Eq 1) and passed to the metabolite PDEs to update the metabolite concentrations at the metabolite grid location where the agent resided using Eq 2. Last in the simulation time step, metabolite diffusion was simulated by solving the continuum-scale PDE models for each metabolite grid (i.e. oxygen, nitrate, NO, glucose).

Metabolite consumption and production time scales were estimated as 0.05 seconds based on metabolite maximum uptake kinetics of oxygen [2]. A general metabolite diffusion time scale, *dt*_*D*_, was estimated as 0.02 seconds based on metabolite diffusion across a one-dimensional patch with length 2 *μm*. Compared to the biomass growth time scale, metabolite reaction and diffusion processes were considered to be at pseudo-steady state, in agreement with previous multi-scale models [59].

When metabolite PDEs were solved, metabolite diffusion was simulated for multiple time steps for every metabolic reaction time step. This approach reconciled the difference between the relatively slow metabolic reaction time scale (0.05s) compared to the fast metabolite diffusion time scale (0.02s), while maintaining stable PDE solutions. The number of times diffusion was performed for carbon and gaseous metabolites was calculated by *dt*_*ex*_/*dt*_*D_carbon*_ and *dt*_*ex*_/*dt*_*D_gas*_, respectively. Accordingly, for carbon substrates with lower magnitude diffusion coefficients and ${dt}_{D_{carbon}}$ similar in magnitude to *dt*_*ex*_, metabolite diffusion was performed 5 times per five-minute simulation time step (Table 2). To achieve stable diffusion coefficients for gases with relatively high magnitude diffusion coefficients and ${dt}_{D_{gas}}$ lower in magnitude compared to *dt*_*ex*_, metabolite diffusion was performed 20 times per five-minute simulation time step.

An experimentally observed growth lag phase was estimated as ~1.5 hours [5] which translated to ~20 five-minute simulation time steps. Therefore, MiMICS simulations were run for 100 five-minute simulation time steps, which represented ten hours of PA14 biofilm growth accounting for the experimentally observed growth lag phase (Table 2). At desired time points, MiMICS output each agent’s index, biomass, growth rate, metabolic state assignment number, intracellular metabolic reaction fluxes, spatial coordinates, and local metabolite concentrations. In addition, the metabolite concentrations for each metabolite grid were output.

### Parameterization

The oxygen threshold parameter, [O_2_]_t_, was varied from 0.19–0.23 mM. The mean absolute error between experiment and MiMICS outputs was calculated from 21 replicate simulations performed for each [O_2_]_t_ parameter value. The total MiMICS model error was calculated from the summation of the error from MiMICS predictions between two experimental outputs: the percentage of denitrification classified cells, and the percentage of oxidative stress classified cells. Denitrification cells were classified to have expression of one or more the following denitrification genes: *nirS*, *norB*, *nosZ*, and no expression of the oxidative stress *katA* gene. Oxidative stress cells were classified to have expression of the oxidative stress *katA* gene. The [O_2_]_t_ parameter value with the smallest error relative to the experiment was used for subsequent simulations.

### MiMICS specifications and code availability

In MiMICS, a Py4J JavaGateway Server was implemented to interface the Java-based HAL platform and Python-based metabolic model. Through this Py4J JavaGateway, functions are called from Python to initialize and run the ABM. Additionally, Python functions called through the JavaGateway retrieve and pass information between Python and Java and save spatial agent and metabolite information at each simulation time point. MiMICS was initially developed on a Mac computer (macOS Catalina v.10.15.7, 1.1 GHz Dual-Core Intel Core i3) using IntelliJ IDEA and 1–2 central processing units (CPUs). All MiMICS simulations were executed on Rivanna High-Performance Computing (HPC) System at the University of Virginia. MiMICS Java files were compiled as a JAR file to run on the Rivanna HPC System. To reduce simulation runtime, MiMICS split metabolic model calculations for each agent across 35 CPUs using Python multiprocessing v.0.70.14. One MiMICS simulation of the ten-hour biofilm growth period on Rivanna HPC System across 35 CPUs took 40 minutes and required 40 GB of memory. When appropriate, multiple MiMICS simulations (e.g. for simulation replicates, parameter sweeps) were executed across multiple computing nodes to prevent multiple JavaGateway servers running on a single computing node. MiMICS simulations used Python v.3.9, cobra v.0.29.0, py4j v.0.10.9.7, Java v.1.8.0, HAL v.1.1.0. MiMICS source code and detailed user guide can be found at: https://github.com/tracykuper/mimics.

## Image analysis and data visualization

Biofilm image analysis of MiMICS simulation outputs with and without motility was performed using Python’s scikit-image (v.0.19.0) regionprops function to measure total biofilm area and average cluster area. Experimental and simulation data and 2D images were plotted with the matplotlib python package (v.3.6.3). 3D images were plotted with plotly package (v.4.14.3)

### Statistical analysis

Statistical analysis was performed with the scipy stats python package (v.1.10.0). Variation among biofilm structure metrics among experimental and simulation conditions was performed with analysis of variance. Pairwise comparisons of individual group means were performed using a Tukey post hoc analysis. Values of *p* < 0.05 were considered statistically significant.

### Spatial neighborhood analysis

Bulk neighborhood analysis of simulation data was performed similarly to the experimental neighborhood analysis [5]. For each gene of interest, agents with active flux encoded by the reaction in the 99^th^ percentile were selected as “center” agents. Using the 3D centroid agent coordinates, distances between center agents and nearby agents within 3 *μm* were calculated. The closest five agents to the center agent were selected as neighbor agents. Neighbor agents were grouped together and the average reaction flux of the neighbors was calculated. This average neighbor reaction flux was divided by the corresponding population average reaction flux, omitting the center agents in the population. For each pair of genes, a Pearson correlation was calculated to determine spatially correlated genes among 50 simulation replicates.

## Supporting information

S1 Fig Unique transcriptomic-guided ten hour and thirty-five hour PA14 biofilm metabolic model states generated from gene expression data extracted using UMAP Leiden clustering method. (A) Joint UMAP cluster analysis performed with ten-hour and thirty-five hour PA14 biofilm spatial transcriptomics data overlaid with the cluster’s metabolic state assignment. The cluster numbers and metabolic states displayed in the legend correspond to the top 9 UMAP Leiden clusters, which captured the majority (91%) of cells in the experimental data. Bar graph quantifies the percentage of the total number of 10-hour and 35-hour biofilm cells within each UMAP Leiden cluster, numbers on the x-axis correspond to the UMAP Leiden cluster number. Shown is also the UMAP overlaid with *pilA* gene expression. (B) For the UMAP Leiden clusters that captured 91% of all biofilm cells, this table categorizes the cluster according to biofilm growth time point and metabolic model state group. Each cluster’s metabolic model state was grouped by metabolic similarity with other models by comparing (C) the similarity of non-consensus reactions and (D) extracellular metabolite flux predictions. Reported are exchange flux values normalized to the maximum exchange flux value among PA14 metabolic models. Experimental data and UMAP analysis methods was provided from Dar and co-workers.
(TIF)

S2 Fig Transcriptome-guided metabolic model states of denitrification PA14 biofilm subpopulations predicted NO-secretion rates and oxidative stress metabolic functions. (A) Percentages of experimental denitrification-classified cells expressing nitrate reductases encoded by *narG* and *napA*. (B) Percentages of experimental denitrification-classified cells with high expression of *narG* and *napA* further classified with and without limiting expression of a gene in the denitrification pathway. (C) Predicted biomass and denitrification pathway exchange fluxes from transcriptome-guided metabolic models of *narG*-expressing denitrification cells and oxidative stress expressing cells. (D) Predicted reaction fluxes of the oxidative stress metabolic model state in varying NO concentrations. (E) Constrained on replete SCFM, predicted growth rates from the four PA14 metabolic model states used in MiMICS. An experimental growth rate was obtained from a *P*. *aeruginosa* aqueous culture in SCFM [29]. Experimental data was provided from Dar and co-workers.
(TIF)

S3 Fig Recapitulating experimental biofilm structure required surface motility ABM rules. Representative *xy* projections and *xz* slices of PA14 biofilms grown for ten hours are shown for (A) experiment and transcriptome-free MiMICS simulations performed (B) with (*pilA*) and (C) without (Δ*pilA*) surface motility ABM rules. Cells plotted in the *xy* projection are located near the z = 0 *μm* surface. Cells are colored with high *pilA* expression. Scale bar represents 20 *μm*. Average (D) total cell count, (E) % area coverage, and (F) cell cluster area comparison between experiment and simulations. Error bars represent one standard deviation of n = 7 experimental replicates and n = 20 simulation replicates. Asterisks represent statistical significance (* p < 0.05, ** p < 0.01, *** p < 0.001). Experimental data was provided by Dar and co-workers.
(TIF)

S4 Fig MiMICS error results to parameterize [O_2_]_t_. Heatmap MiMICS error results for manual parameterization of the oxygen threshold parameter, [O_2_]_t_, in transcriptome-guided MiMICS simulations. [O_2_]_t_ was varied from 0.19–0.23 m*M*. The nitric oxide threshold parameter, [NO]_t_, was held constant at 1 *μM*. MiMICS error is reported from 21 replicate simulations for each [O_2_]_t_ parameter value condition.
(TIF)

S5 Fig Transcriptome-guided MiMICS improved predictions of microscale metabolic heterogeneity and nitric oxide microenvironments in PA14 biofilm compared to transcriptome-free MiMICS. Plotted are *xy* projections of experimental and simulation ten hour PA14 biofilms. Agents are colored accordingly to reaction flux encoded by a gene. Plotted are corresponding *xy* projections of oxygen, nitrate, nitric oxide, and glucose concentration profiles predicted from MiMICS simulations. Note only the transcriptome-guided MiMICS simulation predicted a NO biofilm microenvironment. Scale bar represents 20 *μ*m. Experimental data was reconstructed from Dar and co-workers.
(TIF)

S6 Fig MiMICS captured majority of spatial relationships of intracellular metabolism in PA14 biofilm, but not spatial relationships related to *napA*, encoding a nitrate reductase. Representative *xy* projections of experimental and simulation PA14 biofilms labeled by *napA*, *nirS*, *norB*, and *nosZ* gene expression (experiment) or reaction flux encoded by the respective gene (simulation). Cells plotted are located near the z = 0 *μm* surface. Circled areas highlight regions of interest (ROIs) where denitrification (*nirS*, *norB*, *nosZ*), fermentation, and oxidative stress genes are correlated with one another, and all anticorrelated with the TCA cycle. For example, the *sucC* TCA cycle gene was expressed in fewer cells inside the ROI compared to outside the ROI; while the opposite trend occurs in the expression of denitrification and oxidative stress genes. In addition, the ROIs highlight MiMICS prediction discrepancies of *napA* expression. Specifically, MiMICS predicted *napA*-encoded reaction flux highly localized inside only the ROI, whereas the experiment did not observe large differences of *napA* expression inside and outside of the experimental ROI. Scale bar represents 20 *μm*. Experimental data was reconstructed from Dar and co-workers.
(TIF)

S7 Fig MiMICS computational performance. (A) MiMICS simulation runtime decreased with High Performance Computing (HPC) that split GENRE calculations for each agent across multiple central processing units (CPUs) using parallel processing. Computational runtime reported is from one MiMICS simulation time step. Both CPU conditions were executed on UVA Rivanna HPC. (B) Number of metabolic model states input into MiMICS had a minimal effect on computational runtime. Plotted is the computational time of one MiMICS simulation time step dependent on the number of metabolic model states that an agent could adopt. Simulations were run using 35 CPUs and with 1000 cell agents. Error bars represent one standard deviation from ten replicate simulations.
(TIF)

S8 Fig Updated reactions to PA14 GENRE predicts growth in anaerobic conditions. (A) Predicted biomass growth rates, (B) ubiquinone-9 reaction synthesis flux, and (C) the arginine fermentation reaction predicted by the previous (iPau21) and updated PA14 GENRE. GENREs were simulated in SCFM in aerobic and anaerobic conditions varying in nitrate and L-arginine availability.
(TIF)

S9 Fig Cellular agents dynamically adopting different metabolic states impacts predictions of metabolic state spatial patterns. Representative *xy* image from the parametrized transcriptome-guided MiMICS simulation, which had the ability for agents to dynamically adopt different metabolic states, called the ‘Dynamic metabolic state MiMICS’ here. As a control MiMICS scenario, cells remained fixed in their initialized metabolic state, called the ‘Fixed metabolic state MiMICS’. Dynamic metabolic state MiMICS initialized all cells in the aerobic state. Fixed metabolic state MiMICS initialized cells in an aerobic (2 cells), denitrification (1 cell), denitrification + NO (1 cell), or oxidative stress (1 cell) state. Cells are colored according to metabolic state. Representative *xy* image is shown from a ‘Fixed metabolic state MiMICS’ simulation output. Cells plotted are at the z = 0 *μm* surface. Scale bar represents 20 *μ*m.
(TIF)

S1 Table Gene classification based on overlap if measured in experiment and existence in PA14 GENRE. Genes are classified to overlap if they were measured in spatial transcriptomic experiment and exist in PA14 GENRE.
(DOCX)

S2 Table SCFM media concentrations. SCFM metabolite concentrations used for lower bounds of metabolite exchange during GENRE contextualization and to initialize metabolite patch concentrations in ABM.
(DOCX)

## References

[1] Borriello G, Werner E, Roe F, Kim AM, Ehrlich GD, Stewart PS. Oxygen Limitation Contributes to Antibiotic Tolerance of Pseudomonas aeruginosa in Biofilms. Antimicrobial Agents and Chemotherapy. 2004 Jul 1;48(7):2659–64. doi: 10.1128/AAC.48.7.2659-2664.2004 15215123 PMC434183

[2] Wessel AK, Arshad TA, Fitzpatrick M, Connell JL, Bonnecaze RT, Shear JB, et al. Oxygen Limitation within a Bacterial Aggregate. mBio. 2014 Apr 15;5(2):e00992–14. doi: 10.1128/mBio.00992-14 24736225 PMC3994514

[3] Darch SE, Simoska O, Fitzpatrick M, Barraza JP, Stevenson KJ, Bonnecaze RT, et al. Spatial determinants of quorum signaling in a Pseudomonas aeruginosa infection model. Proc Natl Acad Sci U S A. 2018 May 1;115(18):4779–84. doi: 10.1073/pnas.1719317115 29666244 PMC5939081

[4] Stewart PS, Franklin MJ. Physiological heterogeneity in biofilms. Nat Rev Microbiol. 2008;6(3):199–210. doi: 10.1038/nrmicro1838 18264116

[5] Dar D, Dar N, Cai L, Newman DK. Spatial transcriptomics of planktonic and sessile bacterial populations at single-cell resolution. Science. 2021;373(6556):eabi4882. doi: 10.1126/science.abi4882 34385369 PMC8454218

[6] Ahmed R, Zaman T, Chowdhury F, Mraiche F, Tariq M, Ahmad IS, et al. Single-Cell RNA Sequencing with Spatial Transcriptomics of Cancer Tissues. IJMS. 2022;23(6):3042. doi: 10.3390/ijms23063042 35328458 PMC8955933

[7] Walpole J, Papin JA, Peirce SM. Multiscale Computational Models of Complex Biological Systems. Annu Rev Biomed Eng. 2013;15(1):137–54. doi: 10.1146/annurev-bioeng-071811-150104 23642247 PMC3970111

[8] Biggs MB, Papin JA. Novel Multiscale Modeling Tool Applied to Pseudomonas aeruginosa Biofilm Formation. PLoS ONE. 2013;8(10):e78011. doi: 10.1371/journal.pone.0078011 24147108 PMC3798466

[9] Oberhardt MA, Palsson BØ, Papin JA. Applications of genome-scale metabolic reconstructions. Molecular Systems Biology. 2009;5(1):320. doi: 10.1038/msb.2009.77 19888215 PMC2795471

[10] Becker SA, Palsson BO. Context-Specific Metabolic Networks Are Consistent with Experiments. PLoS Comput Biol. 2008;4(5):e1000082. doi: 10.1371/journal.pcbi.1000082 18483554 PMC2366062

[11] Jenior ML, Moutinho TJ, Dougherty BV, Papin JA. Transcriptome-guided parsimonious flux analysis improves predictions with metabolic networks in complex environments. PLoS Comput Biol. 2020;16(4):e1007099. doi: 10.1371/journal.pcbi.1007099 32298268 PMC7188308

[12] Payne DD, Renz A, Dunphy LJ, Lewis T, Dräger A, Papin JA. An updated genome-scale metabolic network reconstruction of Pseudomonas aeruginosa PA14 to characterize mucin-driven shifts in bacterial metabolism. npj Syst Biol Appl. 2021;7(1):37. 10.1038/s41540-021-00198-2 PMC8501023 34625561

[13] Mahadevan R, Edwards JS, Doyle FJ. Dynamic Flux Balance Analysis of Diauxic Growth in Escherichia coli. Biophysical Journal. 2002;83(3):1331–40. doi: 10.1016/S0006-3495(02)73903-9 12202358 PMC1302231

[14] Wang Y, Ma S, Ruzzo WL. Spatial modeling of prostate cancer metabolic gene expression reveals extensive heterogeneity and selective vulnerabilities. Sci Rep. 2020;10(1):3490. doi: 10.1038/s41598-020-60384-w 32103057 PMC7044328

[15] Karimian E, Motamedian E. ACBM: An Integrated Agent and Constraint Based Modeling Framework for Simulation of Microbial Communities. Sci Rep. 2020;10(1):8695. doi: 10.1038/s41598-020-65659-w 32457521 PMC7250870

[16] Dukovski I, Bajić D, Chacón JM, Quintin M, Vila JCC, Sulheim S, et al. A metabolic modeling platform for the computation of microbial ecosystems in time and space (COMETS). Nat Protoc. 2021;16(11):5030–82. doi: 10.1038/s41596-021-00593-3 34635859 PMC10824140

[17] Bauer E, Zimmermann J, Baldini F, Thiele I, Kaleta C. BacArena: Individual-based metabolic modeling of heterogeneous microbes in complex communities. PLoS Computational Biology. 2017;13(5):e1005544. doi: 10.1371/journal.pcbi.1005544 28531184 PMC5460873

[18] Shlomi T, Eisenberg Y, Sharan R, Ruppin E. A genome-scale computational study of the interplay between transcriptional regulation and metabolism. Molecular Systems Biology. 2007;3(1):101. doi: 10.1038/msb4100141 17437026 PMC1865583

[19] La Rosa R, Johansen HK, Molin S. Adapting to the Airways: Metabolic Requirements of Pseudomonas aeruginosa during the Infection of Cystic Fibrosis Patients. Metabolites. 2019;9(10):234. doi: 10.3390/metabo9100234 31623245 PMC6835255

[20] Bajire SK, Shastry RP. Synergistic effects of COVID-19 and Pseudomonas aeruginosa in chronic obstructive pulmonary disease: a polymicrobial perspective. Mol Cell Biochem. 2023;1–11. doi: 10.1007/s11010-023-04744-w 37129767 PMC10152025

[21] Schwermer CU, de Beer D, Stoodley P. Nitrate respiration occurs throughout the depth of mucoid and non-mucoid Pseudomonas aeruginosa submerged agar colony biofilms including the oxic zone. Sci Rep. 2022;12(1):8557. doi: 10.1038/s41598-022-11957-4 35595796 PMC9123002

[22] Livingston J, Spero MA, Lonergan ZR, Newman DK. Visualization of mRNA Expression in Pseudomonas aeruginosa Aggregates Reveals Spatial Patterns of Fermentative and Denitrifying Metabolism. Appl Environ Microbiol. 2022;88(11):e00439–22. doi: 10.1128/aem.00439-22 35586988 PMC9195945

[23] Su S, Panmanee W, Wilson JJ, Mahtani HK, Li Q, VanderWielen BD, et al. Catalase (KatA) Plays a Role in Protection against Anaerobic Nitric Oxide in Pseudomonas aeruginosa. PLoS ONE. 2014;9(3):e91813. doi: 10.1371/journal.pone.0091813 24663218 PMC3963858

[24] Bravo RR, Baratchart E, West J, Schenck RO, Miller AK, Gallaher J, et al. Hybrid Automata Library: A flexible platform for hybrid modeling with real-time visualization. PLoS Comput Biol. 2020;16(3):e1007635. doi: 10.1371/journal.pcbi.1007635 32155140 PMC7105119

[25] Ebrahim A, Lerman JA, Palsson BO, Hyduke DR. COBRApy: COnstraints-Based Reconstruction and Analysis for Python. BMC Syst Biol. 2013;7(1):74. doi: 10.1186/1752-0509-7-74 23927696 PMC3751080

[26] Jenior ML, Glass EM, Papin JA. Reconstructor: a COBRApy compatible tool for automated genome-scale metabolic network reconstruction with parsimonious flux-based gap-filling. Bioinformatics. 2023;39(6):btad367. doi: 10.1093/bioinformatics/btad367 37279743 PMC10275916

[27] Regulation Arai H. and Function of Versatile Aerobic and Anaerobic Respiratory Metabolism in Pseudomonas aeruginosa. Front Microbio. 2011; 2:103. 10.3389/fmicb.2011.00103 PMC3153056 21833336

[28] Chen F, Xia Q, Ju LK. Aerobic Denitrification of *Pseudomonas aeruginosa* Monitored by Online NAD(P)H Fluorescence. Appl Environ Microbiol. 2003;69(11):6715–22. doi: 10.1128/AEM.69.11.6715-6722.2003 14602632 PMC262322

[29] Palmer KL, Aye LM, Whiteley M. Nutritional Cues Control *Pseudomonas aeruginosa* Multicellular Behavior in Cystic Fibrosis Sputum. J Bacteriol. 2007;189(22):8079–87. 17873029 10.1128/JB.01138-07 PMC2168676

[30] Line L, Alhede M, Kolpen M, Kühl M, Ciofu O, Bjarnsholt T, et al. Physiological levels of nitrate support anoxic growth by denitrification of Pseudomonas aeruginosa at growth rates reported in cystic fibrosis lungs and sputum. Front Microbiol. 2014;5:554. doi: 10.3389/fmicb.2014.00554 25386171 PMC4208399

[31] Wei R, Hui C, Zhang Y, Jiang H, Zhao Y, Du L. Nitrogen removal characteristics and predicted conversion pathways of a heterotrophic nitrification–aerobic denitrification bacterium, Pseudomonas aeruginosa P-1. Environ Sci Pollut Res. 2021;28(6):7503–14. doi: 10.1007/s11356-020-11066-7 33034853

[32] Williams DR, Rowe JJ, Romero P, Eagon RG. Denitrifying Pseudomonas aeruginosa: some parameters of growth and active transport. Appl Environ Microbiol. 1978;36(2):257–63. doi: 10.1128/aem.36.2.257-263.1978 100056 PMC291211

[33] Stewart PS, Zhang T, Xu R, Pitts B, Walters MC, Roe F, et al. Reaction–diffusion theory explains hypoxia and heterogeneous growth within microbial biofilms associated with chronic infections. npj Biofilms Microbiomes. 2016;2(1):16012. doi: 10.1038/npjbiofilms.2016.12 28721248 PMC5515263

[34] De Beer D, Stoodley P, Roe F, Lewandowski Z. Effects of biofilm structures on oxygen distribution and mass transport. Biotech & Bioengineering. 1994;43(11):1131–8. doi: 10.1002/bit.260431118 18615526

[35] Hassan J, Qu Z, Bergaust LL, Bakken LR. Transient Accumulation of NO2- and N2O during Denitrification Explained by Assuming Cell Diversification by Stochastic Transcription of Denitrification Genes. PLoS Comput Biol. 2016;12(1):e1004621. doi: 10.1371/journal.pcbi.1004621 26731685 PMC4701171

[36] Limoli DH, Warren EA, Yarrington KD, Donegan NP, Cheung AL, O’Toole GA. Interspecies interactions induce exploratory motility in Pseudomonas aeruginosa. eLife. 2019;8:e47365. doi: 10.7554/eLife.47365 31713513 PMC6910820

[37] Bartell JA, Blazier AS, Yen P, Thøgersen JC, Jelsbak L, Goldberg JB, et al. Reconstruction of the metabolic network of Pseudomonas aeruginosa to interrogate virulence factor synthesis. Nat Commun. 2017;8:14631. doi: 10.1038/ncomms14631 28266498 PMC5344303

[38] Terasaka E, Yamada K, Wang PH, Hosokawa K, Yamagiwa R, Matsumoto K, et al. Dynamics of nitric oxide controlled by protein complex in bacterial system. Proc Natl Acad Sci USA. 2017;114(37):9888–93. doi: 10.1073/pnas.1621301114 28847930 PMC5603993

[39] Lin YC, Sekedat MD, Cornell WC, Silva GM, Okegbe C, Price-Whelan A, et al. Phenazines Regulate Nap-Dependent Denitrification in Pseudomonas aeruginosa Biofilms. J Bacteriol. 2018;200(9):10–1128. doi: 10.1128/JB.00031-18 29463605 PMC5892114

[40] Vollack KU, Zumft WG. Nitric Oxide Signaling and Transcriptional Control of Denitrification Genes in *Pseudomonas stutzeri*. J Bacteriol. 2001;183(8):2516–26. 11274111 10.1128/JB.183.8.2516-2526.2001 PMC95168

[41] Kuroki M, Igarashi Y, Ishii M, Arai H. Fine-tuned regulation of the dissimilatory nitrite reductase gene by oxygen and nitric oxide in *P* *seudomonas aeruginosa*. Environ Microbiol Rep. 2014 Dec;6(6):792–801. 25186017 10.1111/1758-2229.12212

[42] Robinson JL, Jaslove JM, Murawski AM, Fazen CH, Brynildsen MP. An integrated network analysis reveals that nitric oxide reductase prevents metabolic cycling of nitric oxide by Pseudomonas aeruginosa. Metabolic Engineering. 2017;41:67–81. doi: 10.1016/j.ymben.2017.03.006 28363762

[43] Robinson JL, Brynildsen MP. A Kinetic Platform to Determine the Fate of Nitric Oxide in Escherichia coli. PLoS Comput Biol. 2013;9(5):e1003049. doi: 10.1371/journal.pcbi.1003049 23658508 PMC3642044

[44] Mukhopadhyay P, Zheng M, Bedzyk LA, LaRossa RA, Storz G. Prominent roles of the NorR and Fur regulators in the *Escherichia coli* transcriptional response to reactive nitrogen species. Proc Natl Acad Sci USA. 2004;101(3):745–50. 14718666 10.1073/pnas.0307741100 PMC321752

[45] Barraud N, Schleheck D, Klebensberger J, Webb JS, Hassett DJ, Rice SA, et al. Nitric Oxide Signaling in Pseudomonas aeruginosa Biofilms Mediates Phosphodiesterase Activity, Decreased Cyclic Di-GMP Levels, and Enhanced Dispersal. J Bacteriol. 2009;191(23):7333–42. doi: 10.1128/JB.00975-09 19801410 PMC2786556

[46] Dahal S, Renz A, Dräger A, Yang L. Genome-scale model of Pseudomonas aeruginosa metabolism unveils virulence and drug potentiation. Commun Biol. 2023;6(1):165. doi: 10.1038/s42003-023-04540-8 36765199 PMC9918512

[47] Vo CDT, Michaud J, Elsen S, Faivre B, Bouveret E, Barras F, et al. The O2-independent pathway of ubiquinone biosynthesis is essential for denitrification in Pseudomonas aeruginosa. Journal of Biological Chemistry. 2020;295(27):9021–32. doi: 10.1074/jbc.RA120.013748 32409583 PMC7335794

[48] Meganathan R. Ubiquinone biosynthesis in microorganisms. FEMS Microbiology Letters. 2001;203(2):131–9. doi: 10.1111/j.1574-6968.2001.tb10831.x 11583838

[49] Lu CD, Yang Z, Li W. Transcriptome Analysis of the ArgR Regulon in *Pseudomonas aeruginosa*. J Bacteriol. 2004;186(12):3855–61. 15175299 10.1128/JB.186.12.3855-3861.2004 PMC419968

[50] Diggle SP, Whiteley M. Microbe Profile: Pseudomonas aeruginosa: opportunistic pathogen and lab rat. Microbiology. 2020;166(1):30–3. doi: 10.1099/mic.0.000860 31597590 PMC7273324

[51] Carabelli AM, Isgró M, Sanni O, Figueredo GP, Winkler DA, Burroughs L, et al. Single-Cell Tracking on Polymer Microarrays Reveals the Impact of Surface Chemistry on *Pseudomonas aeruginosa* Twitching Speed and Biofilm Development. ACS Appl Bio Mater. 2020;3(12):8471–80. 10.1021/acsabm.0c00849 PMC8291582 34308271

[52] Golding I, Paulsson J, Zawilski SM, Cox EC. Real-Time Kinetics of Gene Activity in Individual Bacteria. Cell. 2005;123(6):1025–36. doi: 10.1016/j.cell.2005.09.031 16360033

[53] Stewart PS. Diffusion in Biofilms. Journal of Bacteriology. 2003;185(5):1485–91. doi: 10.1128/JB.185.5.1485-1491.2003 12591863 PMC148055

[54] Zacharia IG, Deen WM. Diffusivity and Solubility of Nitric Oxide in Water and Saline. Ann Biomed Eng. 2005;33(2):214–22. doi: 10.1007/s10439-005-8980-9 15771275

[55] Anyan ME, Amiri A, Harvey CW, Tierra G, Morales-Soto N, Driscoll CM, et al. Type IV pili interactions promote intercellular association and moderate swarming of *Pseudomonas aeruginosa*. Proc Natl Acad Sci USA. 2014;111(50):18013–8. 25468980 10.1073/pnas.1414661111 PMC4273417

[56] Davis B, Dulbecco R, Eisen H, Ginsberg H. Bacterial Physiology. In: Microbiology. Second Edition. Maryland: Harper and Row; 1973. p. 96–7.

[57] Volle CB, Ferguson MA, Aidala KE, Spain EM, Núñez ME. Spring constants and adhesive properties of native bacterial biofilm cells measured by atomic force microscopy. Colloids and Surfaces B: Biointerfaces. 2008;67(1):32–40. doi: 10.1016/j.colsurfb.2008.07.021 18815013

[58] Gusnaniar N, Sjollema J, Jong ED, Woudstra W, Vries J, Nuryastuti T, et al. Influence of biofilm lubricity on shear-induced transmission of staphylococcal biofilms from stainless steel to silicone rubber. Microb Biotechnol. 2017;10(6):1744–52. doi: 10.1111/1751-7915.12798 28771954 PMC5658628

[59] Ofiţeru ID, Bellucci M, Picioreanu C, Lavric V, Curtis TP. Multi-scale modelling of bioreactor–separator system for wastewater treatment with two-dimensional activated sludge floc dynamics. Water Research. 2014;50:382–95. doi: 10.1016/j.watres.2013.10.053 24246170

