# Supplementary figures and images for: Spatial transcriptome-guided multi-scale framework connects P. aeruginosa metabolic states to oxidative stress biofilm microenvironment
Source: PLoS Comput Biol. 2024 Apr 26;20(4):e1012031. doi: 10.1371/journal.pcbi.1012031 (PMC11051585; doi:10.1371/journal.pcbi.1012031)

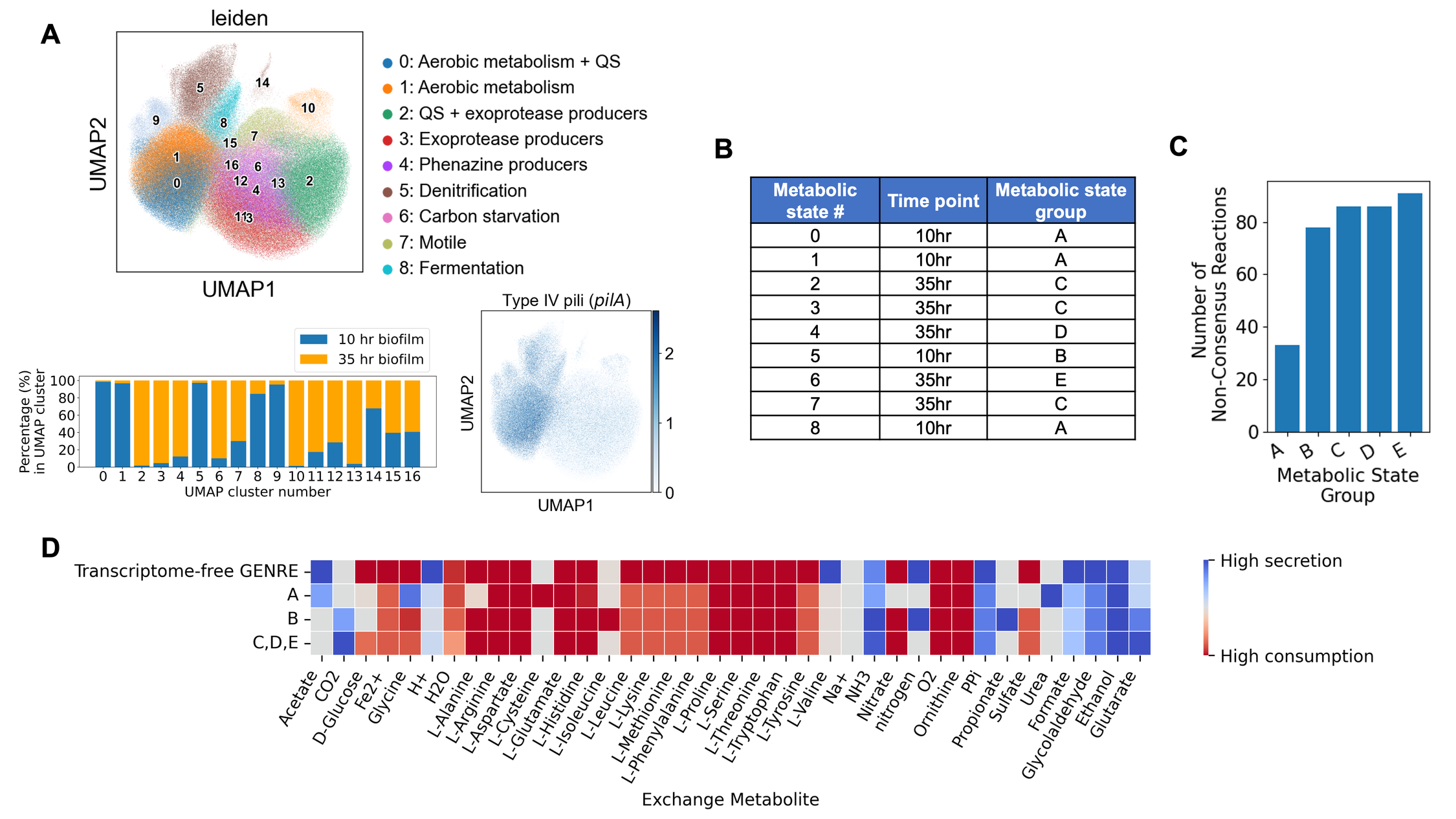

Supplement: S1 Fig — (A) Joint UMAP cluster analysis performed with ten-hour and thirty-five hour PA14 biofilm spatial transcriptomics data overlaid with the cluster’s metabolic state assignment. The cluster numbers and metabolic states displayed in the legend correspond to the top 9 UMAP Leiden clusters, which captured the majority (91%) of cells in the experimental data. Bar graph quantifies the percentage of the total number of 10-hour and 35-hour biofilm cells within each UMAP Leiden cluster, numbers on the x-axis correspond to the UMAP Leiden cluster number. Shown is also the UMAP overlaid with pilA gene expression. (B) For the UMAP Leiden clusters that captured 91% of all biofilm cells, this table categorizes the cluster according to biofilm growth time point and metabolic model state group. Each cluster’s metabolic model state was grouped by metabolic similarity with other models by comparing (C) the similarity of non-consensus reactions and (D) extracellular metabolite flux predictions. Reported are exchange flux values normalized to the maximum exchange flux value among PA14 metabolic models. Experimental data and UMAP analysis methods was provided from Dar and co-workers. (TIF) [file pcbi.1012031.s001.tif]

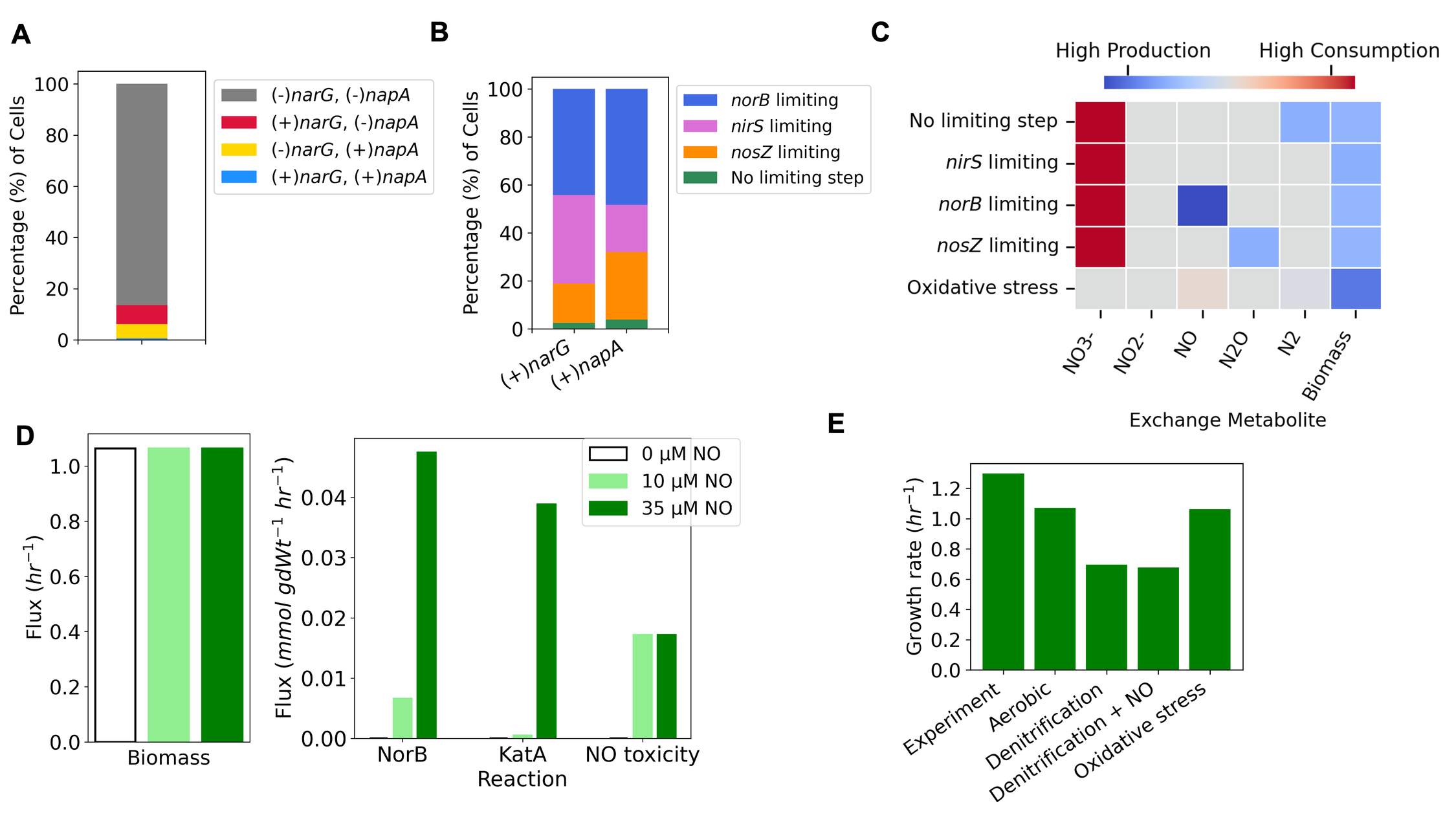

Supplement: S2 Fig — (A) Percentages of experimental denitrification-classified cells expressing nitrate reductases encoded by narG and napA. (B) Percentages of experimental denitrification-classified cells with high expression of narG and napA further classified with and without limiting expression of a gene in the denitrification pathway. (C) Predicted biomass and denitrification pathway exchange fluxes from transcriptome-guided metabolic models of narG-expressing denitrification cells and oxidative stress expressing cells. (D) Predicted reaction fluxes of the oxidative stress metabolic model state in varying NO concentrations. (E) Constrained on replete SCFM, predicted growth rates from the four PA14 metabolic model states used in MiMICS. An experimental growth rate was obtained from a P. aeruginosa aqueous culture in SCFM [29]. Experimental data was provided from Dar and co-workers. (TIF) [file pcbi.1012031.s002.tif]

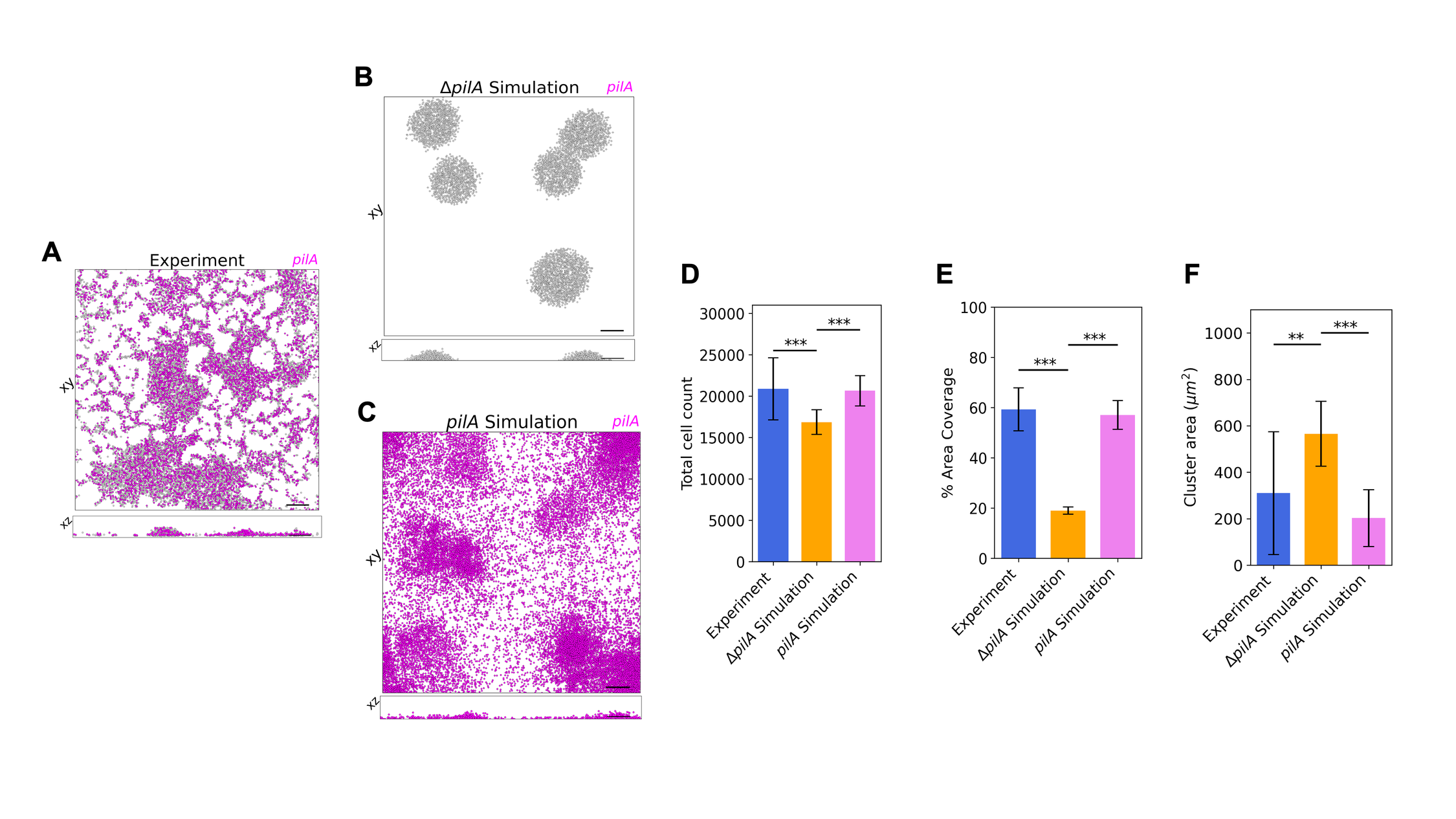

Supplement: S3 Fig — Representative xy projections and xz slices of PA14 biofilms grown for ten hours are shown for (A) experiment and transcriptome-free MiMICS simulations performed (B) with (pilA) and (C) without (ΔpilA) surface motility ABM rules. Cells plotted in the xy projection are located near the z = 0 μm surface. Cells are colored with high pilA expression. Scale bar represents 20 μm. Average (D) total cell count, (E) % area coverage, and (F) cell cluster area comparison between experiment and simulations. Error bars represent one standard deviation of n = 7 experimental replicates and n = 20 simulation replicates. Asterisks represent statistical significance (* p < 0.05, ** p < 0.01, *** p < 0.001). Experimental data was provided by Dar and co-workers. (TIF) [file pcbi.1012031.s003.tif]

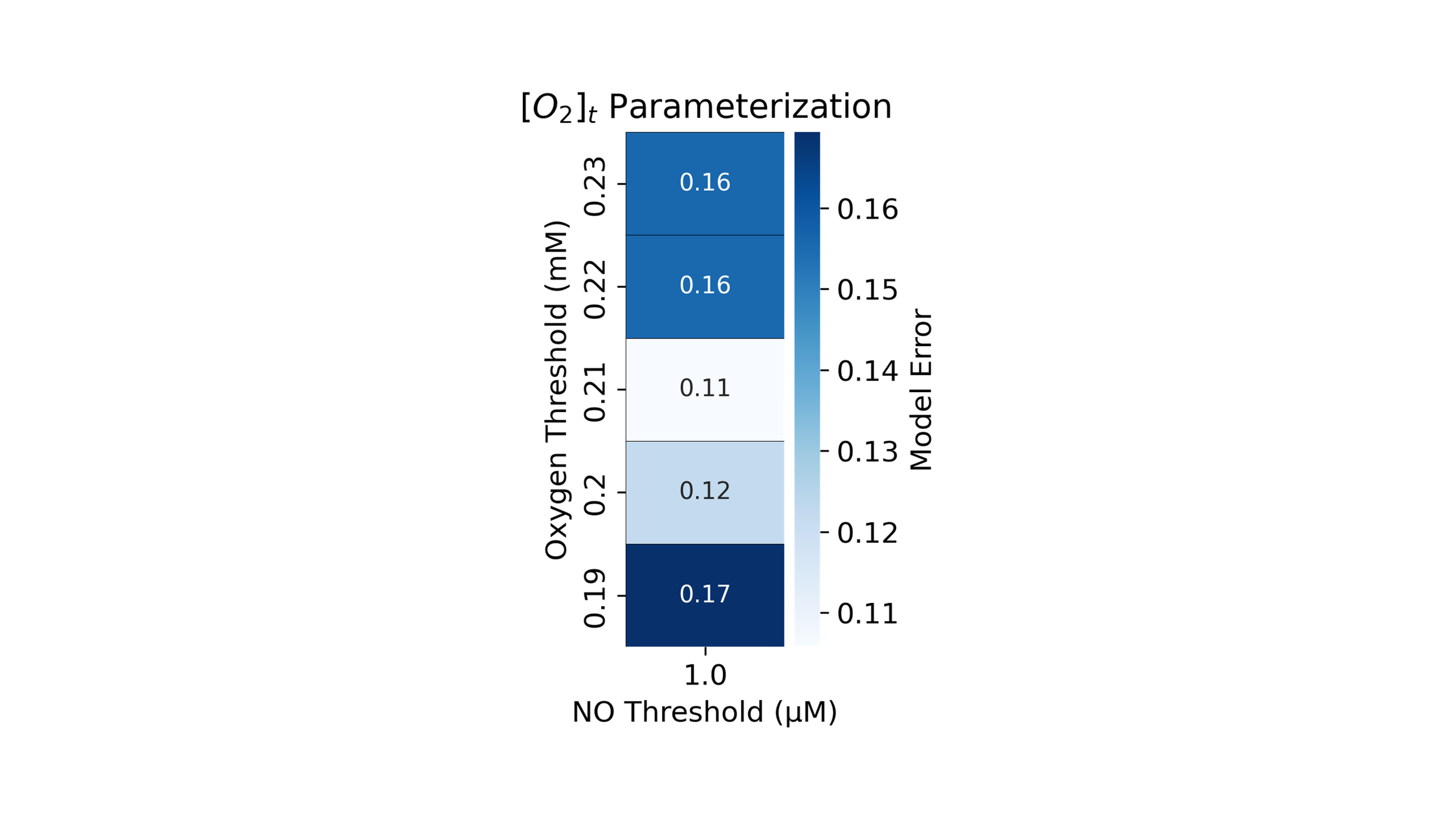

Supplement: S4 Fig — Heatmap MiMICS error results for manual parameterization of the oxygen threshold parameter, [O2]t, in transcriptome-guided MiMICS simulations. [O2]t was varied from 0.19–0.23 mM. The nitric oxide threshold parameter, [NO]t, was held constant at 1 μM. MiMICS error is reported from 21 replicate simulations for each [O2]t parameter value condition. (TIF) [file pcbi.1012031.s004.tif]

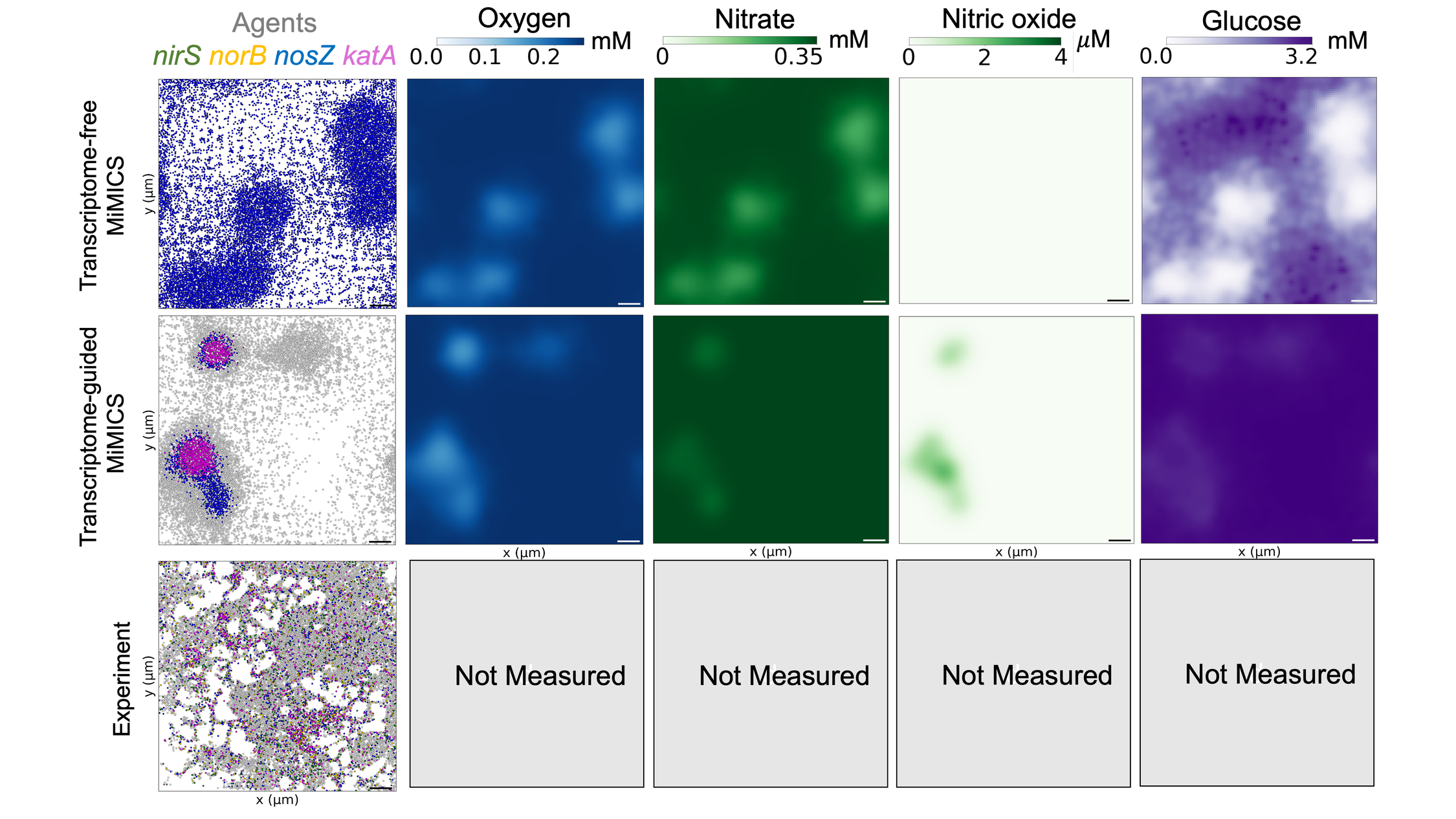

Supplement: S5 Fig — Plotted are xy projections of experimental and simulation ten hour PA14 biofilms. Agents are colored accordingly to reaction flux encoded by a gene. Plotted are corresponding xy projections of oxygen, nitrate, nitric oxide, and glucose concentration profiles predicted from MiMICS simulations. Note only the transcriptome-guided MiMICS simulation predicted a NO biofilm microenvironment. Scale bar represents 20 μm. Experimental data was reconstructed from Dar and co-workers. (TIF) [file pcbi.1012031.s005.tif]

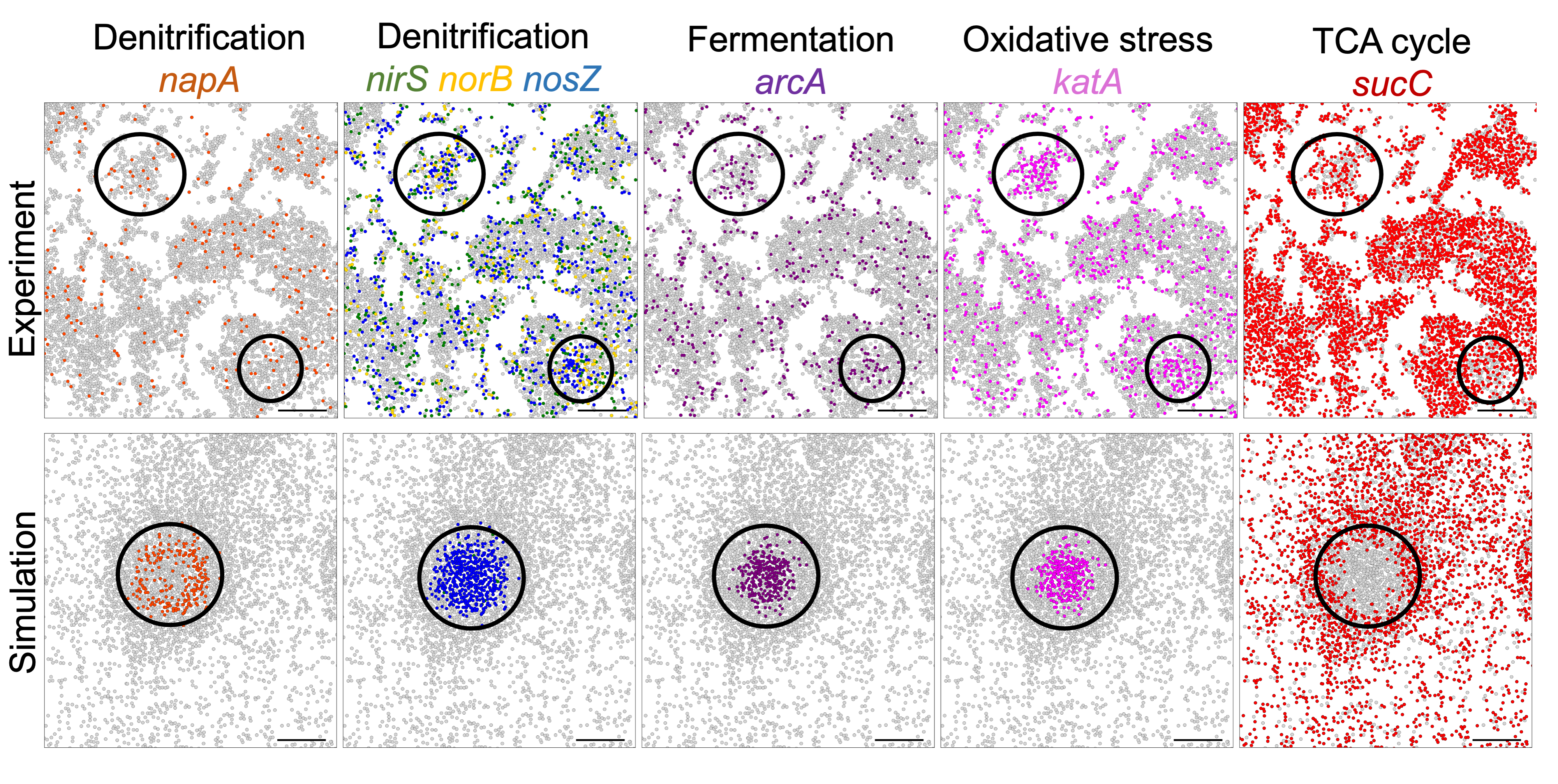

Supplement: S6 Fig — Representative xy projections of experimental and simulation PA14 biofilms labeled by napA, nirS, norB, and nosZ gene expression (experiment) or reaction flux encoded by the respective gene (simulation). Cells plotted are located near the z = 0 μm surface. Circled areas highlight regions of interest (ROIs) where denitrification (nirS, norB, nosZ), fermentation, and oxidative stress genes are correlated with one another, and all anticorrelated with the TCA cycle. For example, the sucC TCA cycle gene was expressed in fewer cells inside the ROI compared to outside the ROI; while the opposite trend occurs in the expression of denitrification and oxidative stress genes. In addition, the ROIs highlight MiMICS prediction discrepancies of napA expression. Specifically, MiMICS predicted napA-encoded reaction flux highly localized inside only the ROI, whereas the experiment did not observe large differences of napA expression inside and outside of the experimental ROI. Scale bar represents 20 μm. Experimental data was reconstructed from Dar and co-workers. (TIF) [file pcbi.1012031.s006.tif]

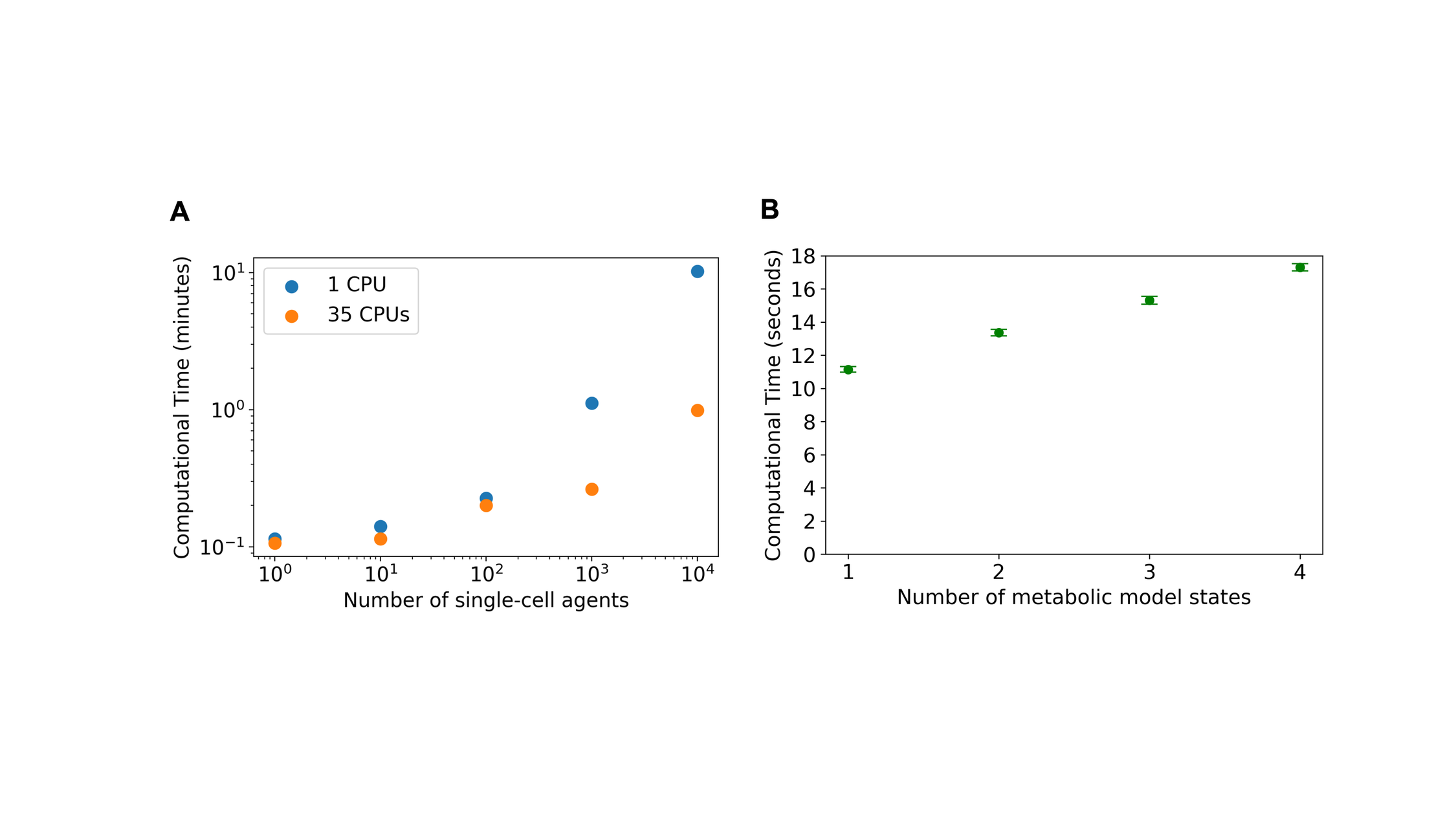

Supplement: S7 Fig — (A) MiMICS simulation runtime decreased with High Performance Computing (HPC) that split GENRE calculations for each agent across multiple central processing units (CPUs) using parallel processing. Computational runtime reported is from one MiMICS simulation time step. Both CPU conditions were executed on UVA Rivanna HPC. (B) Number of metabolic model states input into MiMICS had a minimal effect on computational runtime. Plotted is the computational time of one MiMICS simulation time step dependent on the number of metabolic model states that an agent could adopt. Simulations were run using 35 CPUs and with 1000 cell agents. Error bars represent one standard deviation from ten replicate simulations. (TIF) [file pcbi.1012031.s007.tif]

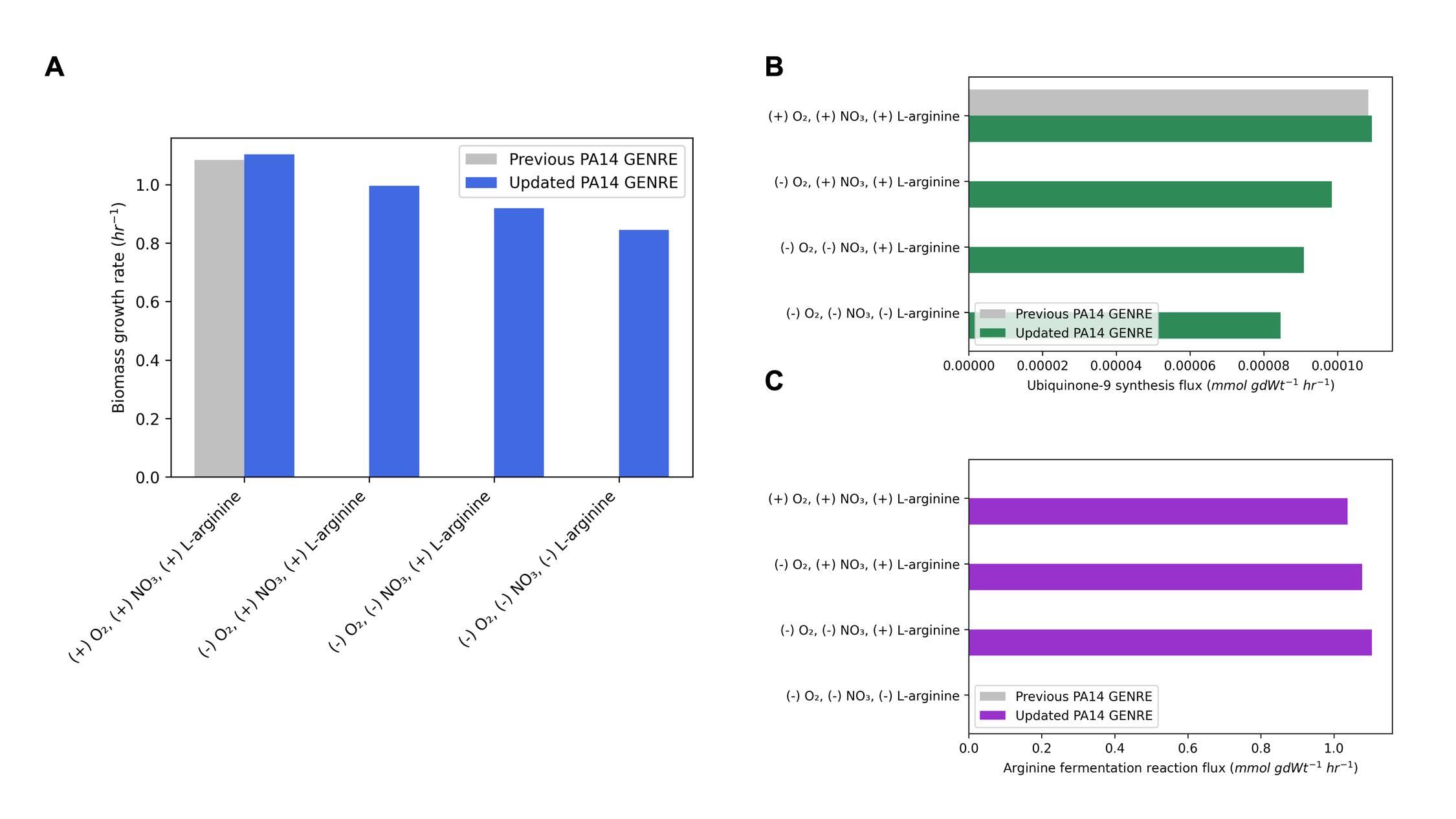

Supplement: S8 Fig — (A) Predicted biomass growth rates, (B) ubiquinone-9 reaction synthesis flux, and (C) the arginine fermentation reaction predicted by the previous (iPau21) and updated PA14 GENRE. GENREs were simulated in SCFM in aerobic and anaerobic conditions varying in nitrate and L-arginine availability. (TIF) [file pcbi.1012031.s008.tif]

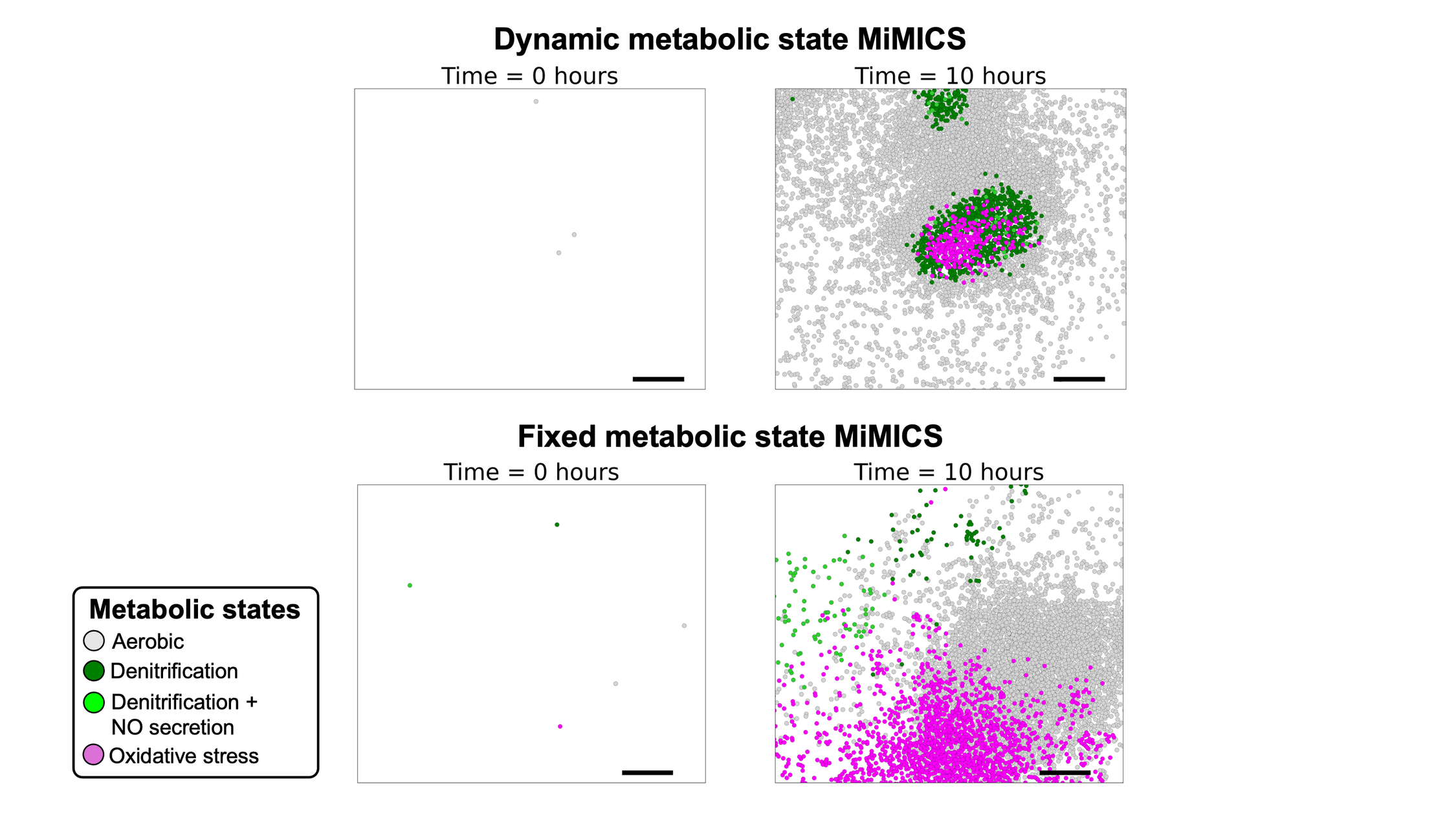

Supplement: S9 Fig — Representative xy image from the parametrized transcriptome-guided MiMICS simulation, which had the ability for agents to dynamically adopt different metabolic states, called the ‘Dynamic metabolic state MiMICS’ here. As a control MiMICS scenario, cells remained fixed in their initialized metabolic state, called the ‘Fixed metabolic state MiMICS’. Dynamic metabolic state MiMICS initialized all cells in the aerobic state. Fixed metabolic state MiMICS initialized cells in an aerobic (2 cells), denitrification (1 cell), denitrification + NO (1 cell), or oxidative stress (1 cell) state. Cells are colored according to metabolic state. Representative xy image is shown from a ‘Fixed metabolic state MiMICS’ simulation output. Cells plotted are at the z = 0 μm surface. Scale bar represents 20 μm. (TIF) [file pcbi.1012031.s009.tif]
