# Supplementary material for: Spatial transcriptome-guided multi-scale framework connects P. aeruginosa metabolic states to oxidative stress biofilm microenvironment
Source: PLoS Comput Biol. 2024 Apr 26;20(4):e1012031. doi: 10.1371/journal.pcbi.1012031 (PMC11051585; doi:10.1371/journal.pcbi.1012031)
Supplement: S1 Table — Genes are classified to overlap if they were measured in spatial transcriptomic experiment and exist in PA14 GENRE. (DOCX) [file pcbi.1012031.s010.docx]

**S1 Table. Gene classification based on overlap if measured in experiment and existence in PA14 GENRE.** Genes are classified to overlap if they were measured in spatial transcriptomic experiment and exist in PA14 GENRE.

| **Gene locus** | **Gene name** | **GENRE overlap** |
| --- | --- | --- |
| P1_gp003 | cre | No |
| P1_gp013 | pro | No |
| P1_gp078 | dbn | No |
| PA14_00010 | dnaA | No |
| PA14_00560 | exoT | No |
| PA14_00640 | phzH | Yes |
| PA14_01160 | vgrG | No |
| PA14_01300 | coxA | No |
| PA14_01720 | ahpF | No |
| PA14_01970 | TriC | No |
| PA14_03650 | cysA | Yes |
| PA14_03920 | spuD | Yes |
| PA14_04410 | ptsP | No |
| PA14_04930 | rpoH | No |
| PA14_05310 | gshB | Yes |
| PA14_05540 | mexB | No |
| PA14_06750 | nirS | Yes |
| PA14_06830 | norB | Yes |
| PA14_06870 | dnr | No |
| PA14_07520 | rpoD | No |
| PA14_08150 | PA14_08150 | No |
| PA14_08370 | vfr | No |
| PA14_08910 | rpsC | No |
| PA14_09115 | rpoA | No |
| PA14_09150 | katA | Yes |
| PA14_09280 | pchF | Yes |
| PA14_09400 | phzS | Yes |
| PA14_09440 | phzE1 | Yes |
| PA14_09490 | phzM | Yes |
| PA14_09520 | mexI | No |
| PA14_10500 | ccoN4 | No |
| PA14_10790 | ampC | Yes |
| PA14_13780 | narG | Yes |
| PA14_14680 | suhB | No |
| PA14_16250 | lasB | No |
| PA14_16500 | wspR | No |
| PA14_17290 | pyrG | Yes |
| PA14_17480 | rpoS | No |
| PA14_17530 | recA | No |
| PA14_18580 | algD | Yes |
| PA14_19100 | rhlA | Yes |
| PA14_19120 | rhlR | No |
| PA14_19130 | rhlI | Yes |
| PA14_20200 | nosZ | Yes |
| PA14_22980 | gltB | Yes |
| PA14_23920 | purF | Yes |
| PA14_24480 | pelA | No |
| PA14_25080 | fadB | Yes |
| PA14_25560 | rne | No |
| PA14_27480 | htpX | No |
| PA14_30050 | aceA | Yes |
| PA14_30630 | pqsH | Yes |
| PA14_32390 | mexF | No |
| PA14_33700 | pvdF | Yes |
| PA14_34050 | impC | Yes |
| PA14_35670 | pslG | No |
| PA14_36310 | hcnC | Yes |
| PA14_36345 | exoY | Yes |
| PA14_38410 | amrB | No |
| PA14_39330 | rbsA | Yes |
| PA14_40290 | lasA | No |
| PA14_40510 | ccoN3 | No |
| PA14_41220 | lon | No |
| PA14_41230 | clpX | No |
| PA14_41440 | uspL | No |
| PA14_41510 | nasA | Yes |
| PA14_41575 | sigX | No |
| PA14_42350 | pscC | No |
| PA14_42500 | pcrD | No |
| PA14_43950 | sucC | Yes |
| PA14_44340 | ccoN2 | No |
| PA14_44370 | ccoN1 | No |
| PA14_44490 | anr | No |
| PA14_45630 | fliA | No |
| PA14_45940 | lasI | Yes |
| PA14_45960 | lasR | No |
| PA14_48060 | aprA | No |
| PA14_48930 | PA14_48930 | No |
| PA14_49250 | napA | Yes |
| PA14_50290 | fliC | No |
| PA14_50360 | flgK | No |
| PA14_51340 | mvfR | No |
| PA14_51410 | pqsC | Yes |
| PA14_52180 | relA | Yes |
| PA14_52270 | ldhA | Yes |
| PA14_52580 | lysC | Yes |
| PA14_53470 | ackA | Yes |
| PA14_54430 | algU | No |
| PA14_56220 | uspM | No |
| PA14_56780 | sodB | Yes |
| PA14_57940 | rpoN | No |
| PA14_58000 | sodM | Yes |
| PA14_58730 | pilA | No |
| PA14_60310 | pilY1 | No |
| PA14_61040 | katB | Yes |
| PA14_61060 | fnr_2 | No |
| PA14_61200 | cdrA | No |
| PA14_62160 | ilvI | Yes |
| PA14_62710 | pnp | No |
| PA14_62730 | truB | Yes |
| PA14_62860 | ftsH | No |
| PA14_68330 | arcA | Yes |
| PA14_69190 | rho | No |
| PA14_70470 | spoT | Yes |
| PA14_70560 | OxyR | No |
| PA14_70860 | pstS | Yes |
| PA14_72960 | kgtP | Yes |
| PA14_73260 | atpA | Yes |
