# Supplementary material for: Spatial transcriptome-guided multi-scale framework connects P. aeruginosa metabolic states to oxidative stress biofilm microenvironment
Source: PLoS Comput Biol. 2024 Apr 26;20(4):e1012031. doi: 10.1371/journal.pcbi.1012031 (PMC11051585; doi:10.1371/journal.pcbi.1012031)
Supplement: S2 Table — SCFM metabolite concentrations used for lower bounds of metabolite exchange during GENRE contextualization and to initialize metabolite patch concentrations in ABM. (DOCX) [file pcbi.1012031.s011.docx]

**S2 Table. SCFM media concentrations.** SCFM metabolite concentrations used for lower bounds of metabolite exchange during GENRE contextualization and to initialize metabolite patch concentrations in ABM.

| **Name** | **Metabolite ID** | **mM in patch (mmol/L)** |
| --- | --- | --- |
| Water | cpd00001_e | 1000 |
| Glucose | cpd00027_e | 3.2 |
| Lactate | cpd00159_e | 9 |
| Alanine | cpd00035_e | 1.8 |
| Arginine | cpd00051_e | 0.3 |
| Aspartate | cpd00041_e | 0.8 |
| Cysteine | cpd00084_e | 0.2 |
| Glutamic acid - glutamate | cpd00023_e | 1.5 |
| Glycine | cpd00033_e | 1.2 |
| histidine | cpd00119_e | 0.5 |
| isoleucine | cpd00322_e | 1.1 |
| leucine | cpd00107_e | 1.6 |
| lysine | cpd00039_e | 2.1 |
| methionine | cpd00060_e | 0.6 |
| phenylalanine | cpd00066_e | 0.5 |
| proline | cpd00129_e | 1.7 |
| ornthinine | cpd00064_e | 0.7 |
| serine | cpd00054_e | 1.4 |
| threonine | cpd00161_e | 1 |
| tryptophan | cpd00065_e | 0.01 |
| tyrosine | cpd00069_e | 0.8 |
| valine | cpd00156_e | 1.1 |
| NH4+ | cpd00013_e | 2.3 |
| SO4 | cpd00048_e | 0.27 |
| Na+ | cpd00971_e | 66.6 |
| HPO4 | cpd00009_e | 2.5 |
| K+ | cpd00205_e | 15.8 |
| Cl- | cpd00099_e | 79.1 |
| Ca2+ | cpd00063_e | 1.7 |
| Mg2+ | cpd00254_e | 0.6 |
| Fe(iii) | cpd00021_e | 0.0036 |
| H+ | cpd00067_e | 2.5 |
| O2 | cpd00007_e | 0.25 |
| CO2 | cpd00011_e | 0.0132 |
| N2 | cpd00528_e | 0.48 |
| Nitrate | cpd00209_e | 0.35 |
